# Horizontal Transfer and Gene Conversion as an Important Driving Force in Shaping the Landscape of Mitochondrial Introns

**DOI:** 10.1534/g3.113.009910

**Published:** 2014-02-10

**Authors:** Baojun Wu, Weilong Hao

**Affiliations:** Department of Biological Sciences, Wayne State University, Detroit, Michigan

**Keywords:** gene conversion, group I intron, horizontal transfer, intron mobility, LSU rRNA, ω intron, mitochondrial genome

## Abstract

Group I introns are highly dynamic and mobile, featuring extensive presence-absence variation and widespread horizontal transfer. Group I introns can invade intron-lacking alleles via intron homing powered by their own encoded homing endonuclease gene (HEG) after horizontal transfer or via reverse splicing through an RNA intermediate. After successful invasion, the intron and HEG are subject to degeneration and sequential loss. It remains unclear whether these mechanisms can fully address the high dynamics and mobility of group I introns. Here, we found that HEGs undergo a fast gain-and-loss turnover comparable with introns in the yeast mitochondrial 21S-rRNA gene, which is unexpected, as the intron and HEG are generally believed to move together as a unit. We further observed extensively mosaic sequences in both the introns and HEGs, and evidence of gene conversion between HEG-containing and HEG-lacking introns. Our findings suggest horizontal transfer and gene conversion can accelerate HEG/intron degeneration and loss, or rescue and propagate HEG/introns, and ultimately result in high HEG/intron turnover rate. Given that up to 25% of the yeast mitochondrial genome is composed of introns and most mitochondrial introns are group I introns, horizontal transfer and gene conversion could have served as an important mechanism in introducing mitochondrial intron diversity, promoting intron mobility and consequently shaping mitochondrial genome architecture.

Group I introns are a special kind of self-splicing ribozyme widely found in protist nuclear ribosomal (r)DNA genes, fungal and plant organellar genomes, bacteria, and viruses (Haugen *et al.* 2005). In eukaryotes, group I introns are highly dynamic, featuring extensive presence-absence variation (Skelly and Maleszka 1991) and widespread horizontal transfer (Colleaux *et al.* 1990; Vaughn *et al.* 1995; Goddard and Burt 1999; Rot *et al.* 2006; Fukami *et al.* 2007). Unrelated group I introns share little conservation at the sequence level, but group I introns mostly contain 10 conserved helices with a structurally conserved catalytic core (Nielsen and Johansen 2009), which is crucial for self-splicing (Adams *et al.* 2004). Group I introns are generally considered neutral to their hosts (Haugen *et al.* 2005) and spread widely thanks to their mobility and to horizontal transfer (Cho *et al.* 1998; Goddard and Burt 1999; Bhattacarya *et al.* 2001; Haugen *et al.* 2005; Sanchez-Puerta *et al.* 2008, 2011). Two main mechanisms are currently recognized for group I intron mobility.

One powerful mechanism for intron mobility is intron homing, powered by active intron-encoded homing endonuclease gene (HEG) (Jacquier and Dujon 1985; Chevalier and Stoddard 2001; Haugen *et al.* 2005; Nielsen and Johansen 2009). During intron homing, HEG initiates a double-strand break repair pathway (Belfort and Roberts 1997; Chevalier and Stoddard 2001) and creates a break at the specific HEG recognition site (around 14−45 nucleotides) within the invaded intronless allele (Colleaux *et al.* 1986). After cleavage, the break is repaired using the intron-containing allele as a template, and the intron and the HEG get integrated as a unit (Lambowitz and Belfort 1993). During group I intron homing, exonic regions immediately flanking the insertion site often engage in a gene conversion process that replaces part of the host exonic sequence and creates a co-conversion tract (CCT) (Mueller *et al.* 1996; Palmer *et al.* 2003; Sanchez-Puerta *et al.* 2011). Goddard and Burt (1999) have proposed a model of the group I intron life cycle that begins with intron homing into an intronless allele followed by intron/HEG degeneration. Once the intron is fixed in the population, the HEG and intron are believed to be on an evolutionary path to loss, and new intron cycles will then start with intron homing in new intronless populations. This intron life cycle model gained support in other genome-wide surveys (Wikmark *et al.* 2006; Milstein *et al.* 2008).

Another mechanism of intron invasion is reverse splicing through an RNA intermediate (Roman *et al.* 1999). A short sequence (4−6 nt) called the internal guide sequence is required for the group I intron to recognize the target region that is complementary to the internal guide sequence, followed by insertion into the transcribed RNA. The group I intron is then incorporated into the genome through reverse transcription of the intron RNA and genomic integration of the resulting cDNA (Roman *et al.* 1999). With much less-specific recognizing nucleotides (4−6 nt) compared with those (14−45 nt) of intron homing, the reverse splicing pathway could permit group I introns to invade not only homologous sites but also heterologous sites (Bhattacharya *et al.* 2002, 2005).

The yeast mitochondrial 21S large subunit (LSU) rRNA contains a group I intron known as the ω intron (Coen *et al.* 1970). The presence of the ω intron within the mitochondrial LSU rRNA has been found to be highly variable among yeast species (Skelly and Maleszka 1991; Goddard and Burt 1999). In this study, we used quantitative and phylogenetic approaches to examine the gain and loss of the ω intron and its HEG sequences from 29 strains in the *Saccharomyces* complex. We have examined the gain and loss of the HEG only among the intron-containing strains, since no existing theories suggest a high frequency of gains and losses of the HEG within the ω intron, and the turnover rate of HEG within the intron would be expected to be very low compared with that of the mobile intron based on the intron-homing model. To our surprise, we found fast turnover rates of the HEG comparable with those of the well-known mobile intron. We further found extensive mosaic sequences in both the HEG and intron sequences, which is inconsistent with any available mechanisms for group I intron mobility, but suggests recurrent horizontal transfer and gene conversion. This mechanism is believed to play important roles in not only introducing intron sequence diversity but also introducing intron content variation and promoting intron mobility by altering the HEG function during the evolution of group I introns.

## Materials and Methods

### Strains and sequence data

Ten *Torulaspora* yeast strains from four species were obtained from the National Center of Agricultural Utilization Research (Peoria, IL) and Dr. Matthew Goddard (The University of Auckland). Genomic DNA of each strain was extracted from overnight cultures following the procedure described in Lee *et al.* (2012). Polymerase chain reaction amplification was performed to obtain the LSU rRNA ω intron and HEG sequences. The nuclear internal transcribed spacer region (ITS1-5.8S-ITS2), 26S rRNA D1/D2, mitochondrial small subunit rRNA and *cox2* sequences were also obtained to infer organismal relationships. The GenBank accession numbers for the sequences used in the study are listed in Supporting Information, Table S1, and the primers used for PCR amplification and Sanger sequencing are listed in Table S2.

### Phylogenetic analyses

Nucleotide sequences were aligned using a combination of MUSCLE (Edgar 2004) and PRANK (Loytynoja and Goldman 2005); sequence alignments were edited manually with SEAVIEW (Gouy *et al.* 2010). Phylogenetic trees were reconstructed using the RAxML program (Stamatakis 2006) under a GTR+Γ+I substitution model. In each phylogenetic reconstruction, 100 bootstrap iterations were performed. Phylogenetic incongruence was examined by the approximately unbiased test (Shimodaira 2002) implemented in the CONSEL program (Shimodaira and Hasegawa 2001). The two nuclear gene regions (ITS1-5.8S-ITS2, 26S rRNA D1/D2) and two mitochondrial genes (small subunit rRNA, and *cox2*) were used to reconstruct the organismal relationship of the *Saccharomyces* complex. The detailed relationship among the three *S. cerevisiae* genomes [288C (Goffeau *et al.* 1996), YJM789 (Wei *et al.* 2007), and No7 (Akao *et al.* 2011)] was determined by the core genomic regions (3380 genes, 477,080 characters) shared by the published nuclear genomes, using the *Saccharomyces mikatae* genome (Kellis *et al.* 2003) as an outgroup.

### Estimation of the gain and loss rates of introns and HEGs

**Figure 1:**
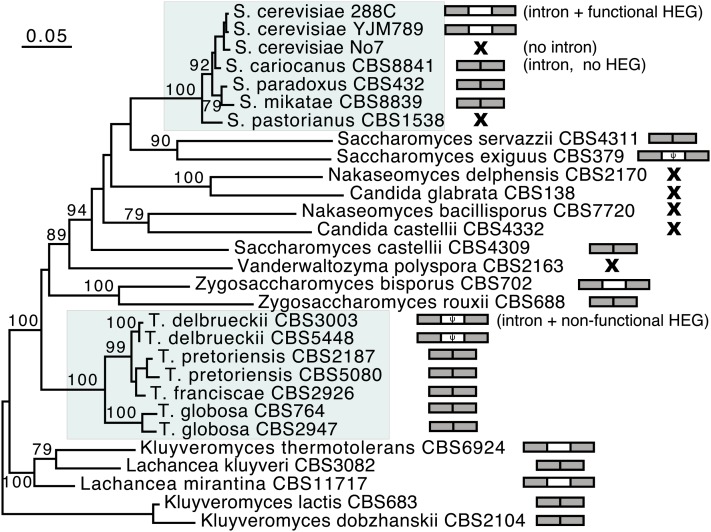
Sporadic distribution of the ω intron and homing endonuclease gene (HEG) in the *Saccharomyces* complex. The phylogenetic relationship was constructed using the concatenated sequences of *cox2*, small subunit rRNA, 26S rRNA D1/D2, and ITS1-5.8S-ITS2. A total of 100 bootstrap iterations were performed, bootstrap values when >70 are shown. The detailed relationship among the three *S. cerevisiae* strains was determined using their core nuclear genomic regions shared with *S. mikatae*. The *Saccharomyces* and *Torulaspora* clades, each of which contains intron or HEG variation among closely related strains, are shaded. The intron-HEG organization of each strain is illustrated after the strain name. Gray boxes and open boxes represent intron and HEG, respectively, whereas crosses stand for intron absence.

We sought to model the gain and loss of an intron or an HEG in homologous sites across organisms related by a phylogeny using a two-state continuous-time Markov process, with states 0 (absence) and 1 (presence). In the *Saccharomyces* complex, to which the *Torulaspora* and *Saccharomyces* genera belong, the phyletic pattern (presence and absence) of the intron and HEG of the LSU rRNA was available in 29 strains. The relationship of these 29 strains (Figure 1) was constructed using the nuclear ITS1-5.8S-ITS2, 26S rDNA D1/D2, mitochondrial small subunit rRNA, and *cox2* genes. The gain and loss rates were estimated using a maximum likelihood estimation implemented in the ACE (ancestral character estimation) function of the APE (analysis of phylogenetics and evolution) package (Paradis *et al.* 2004) in R and BayesTraits (Pagel *et al.* 2004). The estimation was performed separately on the pattern of intron presence/absence within the LSU rRNA of all 29 strains and on the pattern of HEG presence/absence within the intron among the 22 intron-containing strains. The rates of gain and loss in the ω intron and HEG were estimated based on the tree branch lengths and are therefore relative to nucleotide substitution with the unit as the number of gains/losses per site per one nucleotide substitution. Such a concept was developed in Hao and Golding (2006) and has been well received in modeling the rates of gene gain/loss during bacterial genome evolution (Cohen *et al.* 2008; Spencer and Sangaralingam 2009; Hao and Golding 2010; Cohen *et al.* 2011). We found that the two-parameter model separating the gain and loss rates does not significantly outperform the one-parameter model that constrains the rates of gains and losses to be the same on either the intron or HEG data (2Δln*L* < 2.0, *P* > 0.10, df = 1 for either the intron or HEG data, as 2Δln*L* follows approximately a chi-square distribution). Here, we only present the estimations using the simplistic model by constraining the rates of gains and losses to be the same. Furthermore, we have used custom R scripts to compute the likelihood values of given turnover rates in the same way as using the ACE and to conduct likelihood ratio tests between different turnover rates.

## Results and Discussion

### Fast intron turnover and even faster HEG turnover

The presence-absence pattern of the ω intron in the mitochondrial LSU rRNA gene and its encoded HEG was determined in 29 strains within the *Saccharomyces* complex (Figure 1). There are three main intron-HEG organizations: intron with HEG; intron with no HEG; and no intron at all. The HEG sequence in *Torulaspora delbrueckii* CBS3003 was disrupted by a 32-nucleotide high-GC insertion, and the HEG in *T. delbrueckii* CBS5448 was disrupted by a 38-nucleotide high-GC insertion (Figure S1 and Figure S2). The disrupted HEG open reading frames (ORFs) in *T. delbrueckii* were not attributable to sequencing error, as each type of the *T. delbrueckii* sequences represents sequences from at least three different *T. delbrueckii* strains examined in this study (Table S1). The HEGs with an intact ORF were classified as putative functional HEGs, and the ones with a disrupted ORF due to either premature stop codon or indel-associated frame-shift were classified as nonfunctional HEGs. Identical intron-HEG patterns can be found between distantly related strains, whereas very closely related strains may have different intron-HEG organizations. For instance, the ω intron is absent from the *S. cerevisiae* strain No7, whereas both the intron and HEG are present in the other two *S. cerevisiae* strains 288C and YJM789. The *T. delbrueckii* strains contain a copy of non-functional HEG in the intron, while all other *Torulaspora* species only contain an intron with no HEG.

**Figure 2:**
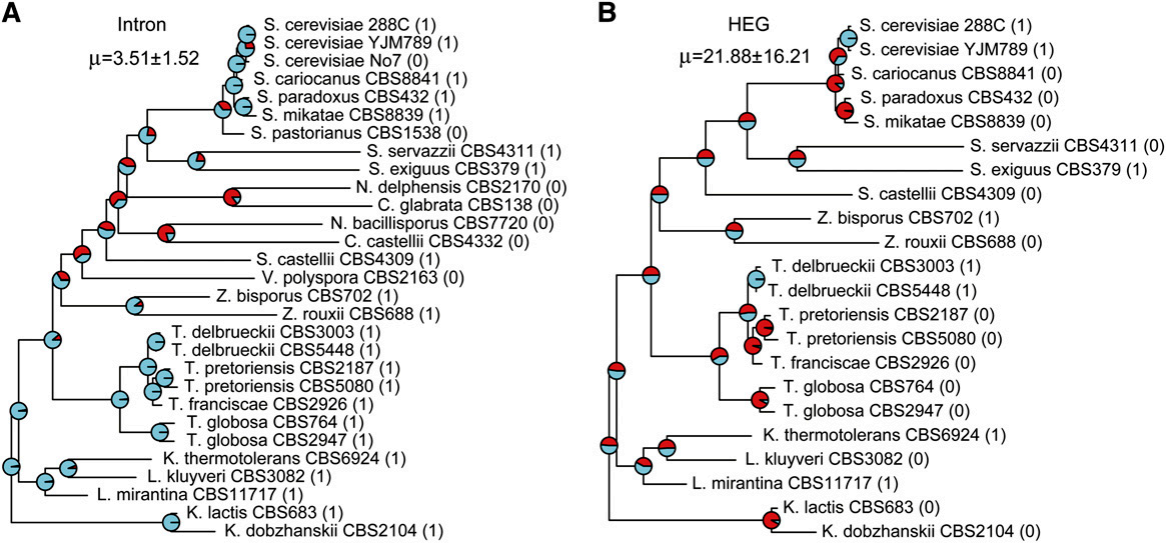
The gain and loss rates of the intron (A) and homing endonuclease gene (HEG) (B) during the evolution of the *Saccharomyces* complex. The phyletic pattern of the intron or HEG is shown in parentheses for each strain. Pie charts illustrate the relative likelihoods (local estimators) of the two possible states (presence or absence) at each ancestral node. An equal rate has been assumed for gains and losses.

To gain a better understanding of the presence-absence variation of the ω intron within the LSU rRNA and the HEG within the ω intron, we sought a quantitative measure for the intron turnover rate and HEG turnover rate. Our analysis assumed equal rates of gains and losses, since the likelihood value of letting the gain and loss rates vary is not significantly better than that of constraining the gain and loss rates to be the same for either the intron or HEG data. Analyses using the ACE function in the Analyses of Phylogenetics and Evolution package (Paradis *et al.* 2004) and BaysianTraits (Pagel *et al.* 2004) gave essentially identical results; here we only present the results from ACE. The intron turnover rate (± SE) was estimated as μ = 3.51 ± 1.52 (Figure 2A), suggesting a faster turnover process of introns than nucleotide substitution. We further calculated the likelihood ratio 2Δln*L* between μ = 3.51 and μ = 1. The latter equals the rate of nucleotide substitution (see *Materials and Methods*), and the estimated intron turnover rate 3.51 is significantly faster than the nucleotide substitution rate (2Δln*L =* 4.26, *P* < 0.05, df = 1). This is in good agreement with the well-known high mobility of group I introns, as ribozymes often are powered by HEGs. Both the intron and its encoded-HEG target the same recognition sequence to promote the mobility of intron/HEG as a unit. If the HEG always goes together with the intron and is lost slowly through mutation accumulation and degeneration, one would expect a very slow HEG turnover rate within the intron. We compared the HEG turnover rate against the intron turnover rate. Please note the null hypothesis here is not that the HEG rate equals the intron turnover rate; instead, the null hypothesis would be that the HEG turnover rate is close to zero if no mechanism promotes the high mobility of HEG within the intron. Surprisingly, however, the turnover rate of HEGs was estimated as μ = 21.88 ± 16.21 (Figure 2B) and is significantly larger than the rate of nucleotide substitution, as 2Δln*L* between μ = 21.88 and μ = 1 is 14.68 (*P* < 0.001, df = 1). On the HEG tree (Figure 2B), the estimated HEG turnover rate of 21.88 is significantly greater than 3.51, the estimated intron turnover rate (2Δln*L =* 4.86, *P* < 0.05, df = 1). A likelihood ratio test between μ = 3.51 and μ = 21.88 was also conducted on the intron tree (Figure 2A), but the likelihood ratio was not significant (2Δln*L=*2.68, *P* > 0.05, df = 1). The different results of the two likelihood ratio tests are likely attributable to the different shapes in their likelihood surfaces (Figure S3). Nevertheless, our results suggest that both the intron and HEG undergo rapid turnover that is faster than the nucleotide substitution rate. The observed trend that the HEG might undergo faster turnover rates than the intron is subject to further investigation in larger datasets.

### High ω intron mobility via intron homing and the unexpectedly high HEG mobility demand an explanation

**Figure 3:**
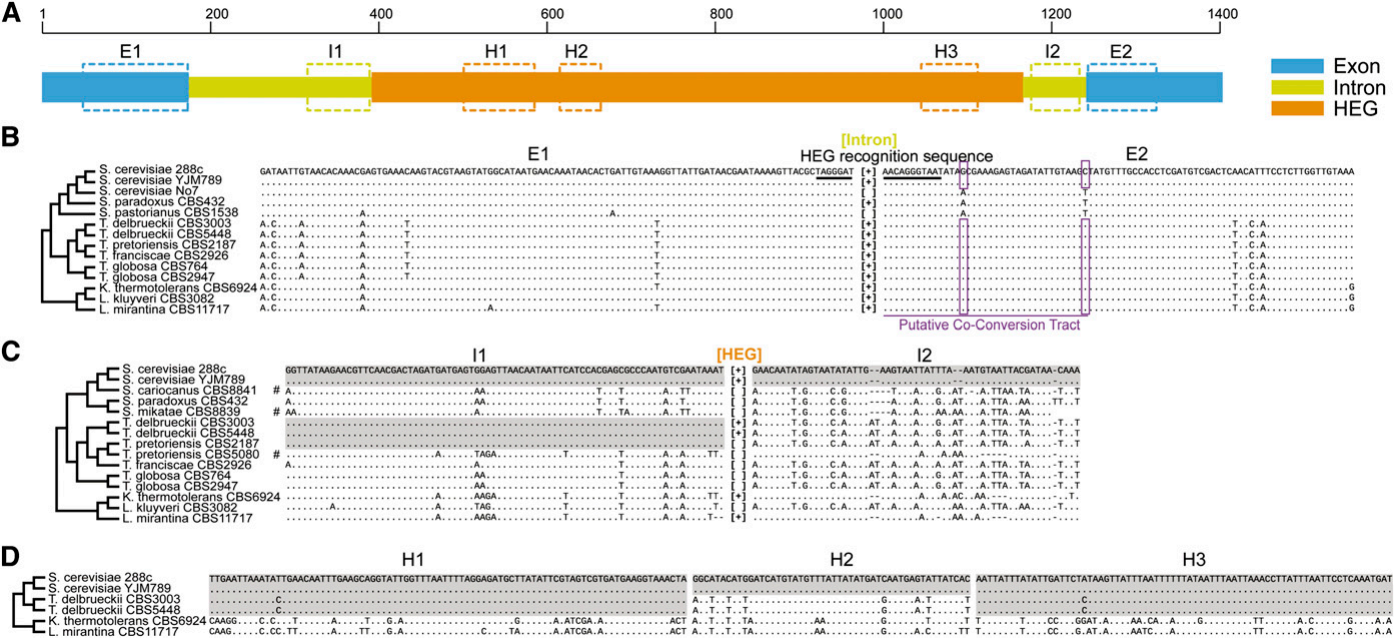
Mosaic intron and homing endonuclease gene (HEG) sequences. (A) An illustration of the large subunit rRNA sequence region examined in the study. Seven small subregions (E1, I1, H1, H2, H3, I2, and E2) were arbitrarily chosen to demonstrate the mosaic nature in detailed sequence alignment. (B) Selected exon regions showing no evidence of gene transfer. Dots indicate identities relative to the *S. cerevisiae* 288C sequence, whereas letters represent nucleotide differences. The 18-nucleotide HEG reorganization sites are underlined. The dendrogram at left was derived from Figure 1. Intron presence is shown as [+], whereas intron absence is shown as [ ]. C) Chimeric structure of the intron sequence. Introns containing HEG are shown as [+], whereas introns lacking HEG are shown as [ ]. Sequences identical with the *S. cerevisiae* 288C sequence are highlighted in gray. (D) Mosaic structure of the HEG sequence. Sequences identical with or differing by only one nucleotide from the *S. cerevisiae* 288C sequence are highlighted in gray.

The HEG within the ω intron has been documented to promote intron insertion by recognizing a specific 18-nucleotide exon sequence (5′-TAGGGATAACAGGGTAAT-3′) (Dujon 1989). We have carefully examined the HEG and intron sequences at a number of subgenic regions (Figure 3, A−D). Among the 22 strains with available exon/intron boundary sequences, 20 strains contain the identical 18-nucleotide HEG recognition sequences (14 strains are shown in Figure 3B). *Nakaseomyces bacillisporus* and *Candida castellii* differed by one nucleotide out of the 18 HEG recognition nucleotides from the rest of the strains (not shown). The highly conserved HEG recognition sequence could have served as an excellent precondition for active ω intron invasion promoted by HEG, which is believed to be highly specific on the target sites (Dujon 1989).

CCTs are considered the footprints of previous invading introns. In this study, the putative CCT spans the nucleotides G and C at sites +15 and +37 downstream from the intron insertion site, whereas the two intron-lacking strains contain nucleotides A and T at the corresponding sites. Group I intron invasion can also take place in the absence of the CCT (Sanchez-Puerta *et al.* 2008). In fact, *S. paradoxus* CBS432 contains the intron but does not have the signature CCT nucleotides G_+15_ and C_+37_ (Figure 3B). In conclusion, intron homing has occurred in most strains in the *Saccharomyces* complex and made important contributions to the high turnover of introns.

Intron homing might well explain the high turnover rate of introns, but it cannot explain the apparently high turnover rate of HEGs. If the mobility of HEGs was primarily due to HEG degeneration and loss by mutation accumulation, the HEG would be present in most introns, as mutation accumulation is a much slower process than the turnover of introns and the HEG turnover rate would be expected to be very low or close to zero as HEG turnover was estimated only among the intron-containing taxa. We then investigated the possibility that the unexpectedly fast turnover rate of HEG could be introduced by artifacts in the analysis. First, an intron/HEG presence-absence polymorphism within a defined species (*e.g.*, *S. cerevisiae* in Figure 1) could result in high estimates for the turnover rate. However, only an intron presence-absence polymorphism was observed among the three *S. cerevisiae* strains, and no HEG presence-absence polymorphism was observed between the two intron-containing *S. cerevisiae* strains. Second, we noticed that our obtained “organismal” relationship based on the concatenated four-gene sequences in Figure 1 slightly differs from the topology published by Kurtzman (2003) (mostly on the low-bootstrap-support branches). To minimize the concern that possible phylogenetic uncertainty might affect rate estimation, we performed maximum likelihood estimation on the Kurtzman-topology by using the same set of taxa (Figure S4). In this case, a similarly fast turnover rate of HEG was observed.

We then considered whether any previously recognized mechanisms could explain the high HEG mobility. HEGs themselves have been suggested to be mobile elements independent of a host intron (Sellem and Belcour 1997). There has been evidence of phylogenetic incongruence between the intron and the intron-encoded-HEG trees among fungal nuclear rDNA, involving intron-independent mobility of HEGs into both homologous and heterologous positions within a group I intron (Haugen and Bhattacharya 2004; Haugen *et al.* 2004). Recent studies have suggested that the intron-encoded HEGs were once free-standing endonucleases and the introns and their HEGs evolved separately to target the same highly conserved sequences, uniting afterward to create a composite mobile element (Bonocora and Shub 2009; Zeng *et al.* 2009). The specificity of the HEG recognition sequence can serve as a guide for detecting intron-independent HEG movements. That is, the flanking sequence at the recent insertion site of a group I intron-encoded HEG is expected to be very similar to the HEG recognition sequence (Loizos *et al.* 1994). In this study, however, no sequences flanking the HEG in the ω intron were found to be similar to the HEG recognition sequence (see Figure S2 for details). The observed high HEG mobility, therefore, could not be explained by its own recent cleavage activity.

HEG degeneration and sequential loss caused by mutation accumulation can contribute to HEG turnover (Goddard and Burt 1999). Our analysis, however, observed HEG turnover rates significantly greater than the nucleotide substitution rate, which could not be explained solely by the mutation-initiated HEG degeneration and loss. Furthermore, reverse splicing is believed to have little impact on the fast turnover of HEG, because the reverse splicing pathway generally involves intron and HEG moving together as a unit (Haugen and Bhattacharya 2004). A satisfactory explanation for the very fast HEG turnover, therefore, demands mechanisms involving genetic changes that are more sudden and/or substantial than mutations.

### Mosaic structure of the ω intron and HEG implicates horizontal transfer and gene conversion

**Figure 4:**
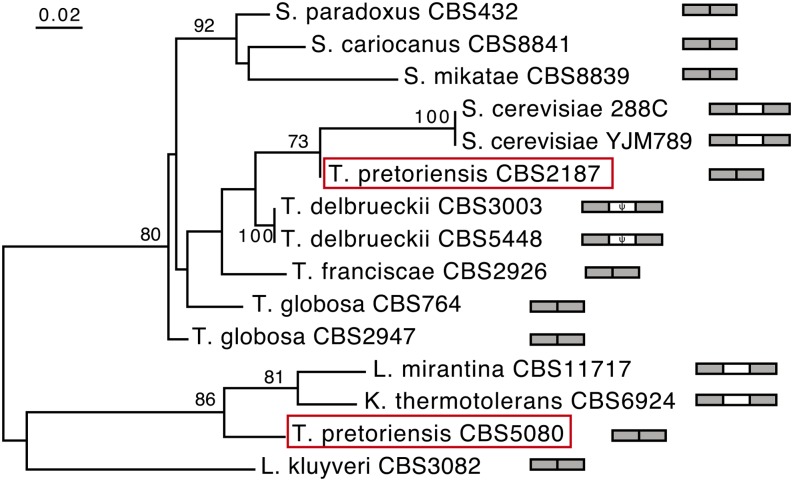
Maximum likelihood tree of the *Saccharomyces*, *Torulaspora*, and *Lachancea* intron sequences in the large subunit rRNA gene. Phylogenetic analysis was based on the sequence alignment shown in Figure S1. Bootstrap values when >60% are shown. The two *T. pretoriensis* strains located at remarkably different phylogenetic positions are boxed. As in Figure 1, gray boxes and open boxes represent intron and homing endonuclease gene, respectively.

Two *T. pretoriensis* strains, CBS2187 and CBS5080, respectively, contain remarkably different intron sequences. *T. pretoriensis* CBS2187 is very similar to the two *T. delbrueckii* strains and *S. cerevisiae*. *T. pretoriensis* CBS5080 is similar to *K. thermotolerans* and *L. mirantina* in different genera. Significant phylogenetic incongruence was observed between the intron tree and organismal tree (*P* < 0.001, approximately unbiased test; Figure 4), indicating horizontal transfer of the intron.

Mosaic intron sequences were found in a number of strains, *i.e.*, different regions within an intron were of different evolutionary origins, which is inconsistent with intron homing or reverse splicing. For instance, *T. pretoriensis* CBS2187 and the two *T. delbrueckii* strains are identical to two *S. cerevisiae* strains 288C and YJM789 in intron region I1 (Figure 3C). In intron region I2, however, they are highly similar (with no more than two nucleotides different) to all other *Torulaspora* strains except *T. pretoriensis* CBS5080, but differed from the *S. cerevisiae* strains by 17 or 18 nucleotide substitutions plus a two-nucleotide indel. The chimeric intron sequences in *T. pretoriensis* CBS2187 and *T. delbrueckii* are believed to be the result of gene conversion after the horizontal transfer of the intron.

On the basis of the I1 and I2 regions, *T. pretoriensis* CBS2187 was found to be remarkably similar to the two *T. delbrueckii* strains. *T. pretoriensis* CBS5080 was strikingly different from *T. pretoriensis* CBS2187 and other *Torulaspora* strains but similar to *K. thermotolerans* and *L. mirantina*. However, these relationships do not hold for the entire intron sequences. For instance, the beginning of the *T. pretoriensis* CBS5080 (alignment positions 169−245 in Figure S1) was identical with *T. delbrueckii* CBS5448, but differed from either *K. thermotolerans* or *L. mirantina* by at least 12 nucleotides plus one indel. In *T. pretoriensis* CBS2187, at least two intron regions (alignment positions 212−304, and 1257−1287 in Figure S2) were different from *T. delbrueckii* but identical with the two *S. cerevisiae* strains. These findings suggest that horizontal transfer and gene conversion can take place recurrently in the intron sequences with multiple donor strains and can be at a fine-scale. We have previously demonstrated that gene conversion between foreign and native homologs can significantly confound phylogenetic analysis (Hao and Palmer 2011), it is not unreasonable to believe that the true evolutionary history of these group I introns could be much more complex than we have inferred.

Like the mosaic intron sequences, the HEG sequences are also highly mosaic (Figure 3D). The H1 (86 nucleotide in length) and H3 (75 nucleotide in length) regions in *T. delbrueckii* were found to differ by only one nucleotide from the two *S. cerevisiae* strains, but the H2 region (50 nucleotides in length) in *T. delbrueckii* differs from the two *S. cerevisiae* strains by eight nucleotides, all of which could be found in either *K. thermotolerans* or *L. mirantina*. It is important to mention that along the *T. delbrueckii* HEG sequence there are additional regions highly similar or identical with *S. cerevisiae*, and some other regions that are significantly different from *S. cerevisiae* but for which the donor species could not be successfully identified (Figure S1). These findings strongly suggest that frequent gene conversion has taken place within the HEG sequence.

We used the PHI (pairwise homoplasy index) package (Bruen *et al.* 2006) to statistically examine the significance of gene conversion. Significant recombination signals (*P* < 0.001, PHI test) were found in both the intron and HEG sequences. In a contrast, no significant recombination signal was detected in the exon sequences, nor was significant phylogenetic incongruence observed between the exon tree and organismal tree (Figure S5). In this study, we focused primarily on the demonstration of the highly mosaic nature of the intron and HEG sequences and did not attempt to identify all the possible gene conversion breakpoints. For illustration purposes, the regions in Figure 3 were arbitrarily chosen, as our previous studies have shown that the recombination breakpoints cannot all be correctly detected by existing recombination detection programs, especially when recombination is frequent and recurrent, (Hao 2010; Hao *et al.* 2010).

### Horizontal transfer and gene conversion alter HEG content and function

All the *Torulaspora* strains, except *T. pretoriensis* CBS5080, share nearly identical intron sequences in the I2 regions, but only the *T. delbrueckii* introns contain HEG (Figure 3C). It is possible that *T. delbrueckii* once had a native HEG-lacking intron just like other *Torulaspora* species. We tend to disfavor the explanation that intron homing introduced HEG within a chimeric intron in *T. delbrueckii*, since if the *T. delbrueckii* ancestor, like other *Torulaspora* species, already had an HEG-lacking intron, the intron would interrupt the HEG recognition sequence and prevent the homing process. Unlike intron homing, gene conversion can explain the gain of HEG associated with the mosaic intron sequence in *T. delbrueckii* (Figure 2C and Figure S2). It is very likely that the *T. delbrueckii* HEG resulted from gene conversion between a native HEG-lacking intron and a foreign *S. cerevisiae*-like HEG-containing intron. It is also noteworthy that the HEG-containing intron in *S. cerevisiae* itself is likely of foreign origin, as the intron sequences in the two *S. cerevisiae* strains are not clustered with their sister species, *S. paradoxus*, *S. cariocanus*, and *S. mikatae*, which formed a phylogenetic clade (Figure 4).

The overall intron sequence in *T. pretoriensis* CBS2187 is remarkably similar to the two *T. delbrueckii* strains, whereas the overall intron sequence in *T. pretoriensis* CBS5080 is similar to *K. thermotolerans* and *L. mirantina*. The *K. thermotolerans*, *L. mirantina* and *T. delbrueckii* strains all contain an HEG in their introns, whereas the two *T. pretoriensis* strains lack the HEG. The mosaic intron sequences within *T. pretoriensis* (Figure S1 and Figure S2) do not support the scenario of HEG loss after homing of an HEG-containing intron because intron homing would have introduced the HEG and intron as a unit from the very same donor. One possible explanation is that horizontal transfer of an HEG-containing intron has taken place independently in each of the two *T. pretoriensis* strains; each foreign HEG-containing intron (*K. thermotolerans-L. mirantina*-like or *T. delbrueckii*-like) has been separately converted to an HEG-lacking intron by the presumably native HEG-lacking intron via gene conversion.

**Figure 5:**
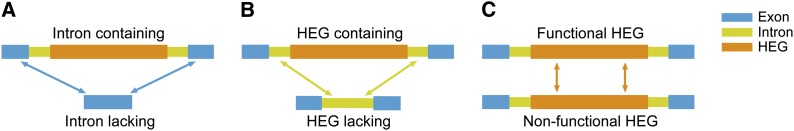
Rapid alteration of mitochondrial intron content via horizontal transfer and gene conversion. (A) Gene conversion that takes place in the exon region can result in the gain or loss of the whole intron. This has been previously reported in the loss of the *cox2* intron in plants (Hepburn *et al.* 2012). (B) Gene conversion that takes place in the intron region can result in chimeric introns, the gain and loss of homing endonuclease gene (HEG) as per Figure 3. (C) Gene conversion that takes place within HEG can result in chimeric HEG, and the alteration between functional HEG and nonfunctional HEG as per Figure 3.

Gene conversion occurs within group I introns independently of the HEG-recognition sequence and HEG function, which therefore increases the chance of genetic exchange. As a consequence, group I introns can show high sequence diversity and high HEG turnover rates. Furthermore, gene conversion also takes place within the HEG sequence, which could potentially alter HEG function in two ways. (1) Gene conversion could pseudogenize a previously functional HEG open reading frame, leading to HEG degeneration, HEG loss, and ultimately intron loss. (2) Gene conversion could rescue a degenerated HEG back to an intact and functional HEG and promote further intron invasion. Together with the gene conversion between the HEG-containing and HEG-lacking introns, gene conversion can be a powerful force driving HEG and intron evolution (Figure 5).

### On the possibility of gene conversion at the exon region

A growing body of evidence has shown that, after horizontal transfer of foreign homologs, gene conversion takes place between the foreign and native homologs to introduce sequence diversity, sequence content variation, and even the gain and loss of adjacent dispensable genes (Bergthorsson *et al.* 2003; Barkman *et al.* 2007; Hao *et al.* 2010; Mower *et al.* 2010; Hepburn *et al.* 2012; Kong *et al.* 2013). It is therefore not unreasonable to suspect that gene conversion can take place at the exon region of a group I intron following horizontal transfer and directly alter intron presence or absence. This study has only discovered evidence of frequent gene conversion in the intron and HEG sequences, not in the exon sequences. We tend to believe that gene conversion occurs at a much higher frequency in the intron and HEG regions than in the exon region, largely because group I introns are likely under less functional constraint than native protein coding sequences. The possibility of gene conversion at the exon regions can only reinforce the importance of horizontal transfer and gene conversion on the turnover of group I introns.

Group I intron invasion is generally believed to introduce the intron as a whole into an intron-less allele. Up to date, few studies questioned the presumption that the whole intron is of a single origin. In this study, our maximum likelihood analysis supports that HEGs undergo faster or at least comparable turnover compared with the intron in the mitochondrial LSU rRNA gene from the *Saccharomyces* complex, which seemed to be incompatible with current working theories on the movement of group I introns. Our sequence analysis discovered evidence of recurrent gene conversion within the intron and HEG following horizontal transfer. These findings suggest that frequent horizontal transfer and gene conversion can alter HEG content within a group I intron (Figure 5), rescue the nonfunctional HEG, and avoid the ultimate fate of HEG loss and intron loss. Thus, horizontal transfer and gene conversion can play an important role in promoting group I intron mobility via the change of HEG content and HEG sequence. Given the abundance of group I introns in fungal mitochondrial genomes, horizontal transfer and gene conversion would play a significant role in shaping mitochondrial genome architecture. Our results are consistent with the increasingly appreciated role of gene conversion on mitochondrial genome evolution. For instance, gene conversion has recently been shown to take place between the two ends of linear mitochondrial genomes and shapes linear mitochondrial genome architecture (Smith and Keeling 2013). Our conclusions on a mitochondrial group I intron in this study could have a broad implication that gene conversion within a mobile intron can alter the presence/absence and the function of an endonuclease or a retrotranscriptase and ultimately promote the gain and loss of the mobile intron. This might not only be true in organellar genomes, but might also be true in nuclear genomes (*e.g.*, group I, II introns, transposable elements). Considering the wide spread of mobile introns and elements, horizontal transfer and gene conversion could have a significant impact on eukaryotic genomes. All of this could be tested using fast growing genomic data.

## Supplementary Material

Supporting Information

## References

[bib1] Adams P. L. Stahley M. R. Kosek A. B. Wang J. Strobel S. A., 2004 Crystal structure of a self-splicing group I intron with both exons. Nature 430: 45–50. 15175762 10.1038/nature02642

[bib2] Akao T. Yashiro I. Hosoyama A. Kitagaki H. Horikawa H., 2011 Whole-genome sequencing of sake yeast *Saccharomyces cerevisiae* Kyokai no. 7. DNA Res. 18: 423–434. 21900213 10.1093/dnares/dsr029 PMC3223075

[bib3] Barkman T. J. McNeal J. R. Lim S. H. Coat G. Croom H. B., 2007 Mitochondrial DNA suggests at least 11 origins of parasitism in angiosperms and reveals genomic chimerism in parasitic plants. BMC Evol. Biol. 7: 248. 18154671 10.1186/1471-2148-7-248 PMC2234419

[bib4] Belfort M. Roberts R. J., 1997 Homing endonucleases: keeping the house in order. Nucleic Acids Res. 25: 3379–3388. 9254693 10.1093/nar/25.17.3379 PMC146926

[bib5] Bergthorsson U. Adams K. L. Thomason B. Palmer J. D., 2003 Widespread horizontal transfer of mitochondrial genes in flowering plants. Nature 424: 197–201. 12853958 10.1038/nature01743

[bib6] Bhattacarya D. Cannone J. J. Gutell R. R., 2001 Group I intron lateral transfer between red and brown algal ribosomal RNA. Curr. Genet. 40: 82–90. 11570520 10.1007/s002940100227

[bib7] Bhattacharya D. Friedl T. Helms G., 2002 Vertical evolution and intragenic spread of lichen-fungal group I introns. J. Mol. Evol. 55: 74–84. 12165844 10.1007/s00239-001-2305-x

[bib8] Bhattacharya D. Reeb V. Simon D. M. Lutzoni F., 2005 Phylogenetic analyses suggest reverse splicing spread of group I introns in fungal ribosomal DNA. BMC Evol. Biol. 5: 68. 16300679 10.1186/1471-2148-5-68 PMC1299323

[bib9] Bonocora R. P. Shub D. A., 2009 A likely pathway for formation of mobile group I introns. Curr. Biol. 19: 223–228. 19200727 10.1016/j.cub.2009.01.033 PMC2856452

[bib10] Bruen T. C. Philippe H. Bryant D., 2006 A simple and robust statistical test for detecting the presence of recombination. Genetics 172: 2665–2681. 16489234 10.1534/genetics.105.048975 PMC1456386

[bib11] Chevalier B. S. Stoddard B. L., 2001 Homing endonucleases: structural and functional insight into the catalysts of intron/intein mobility. Nucleic Acids Res. 29: 3757–3774. 11557808 10.1093/nar/29.18.3757 PMC55915

[bib12] Cho Y. Qiu Y. L. Kuhlman P. Palmer J. D., 1998 Explosive invasion of plant mitochondria by a group I intron. Proc. Natl. Acad. Sci. USA 95: 14244–14249. 9826685 10.1073/pnas.95.24.14244 PMC24358

[bib13] Coen D. Deutsch J. Netter P. Petrochilo E. Slonimski P. P., 1970 Mitochondrial genetics. I. Methodology and phenomenology. Symp. Soc. Exp. Biol. 24: 449–496. 5516343

[bib14] Cohen O. Rubinstein N. D. Stern A. Gophna U. Pupko T., 2008 A likelihood framework to analyse phyletic patterns. Philos. Trans. R. Soc. Lond. B Biol. Sci. 363: 3903–3911. 18852099 10.1098/rstb.2008.0177 PMC2607420

[bib15] Cohen O. Gophna U. Pupko T., 2011 The complexity hypothesis revisited: connectivity rather than function constitutes a barrier to horizontal gene transfer. Mol. Biol. Evol. 28: 1481–1489. 21149642 10.1093/molbev/msq333

[bib16] Colleaux L. d’Auriol L. Betermier M. Cottarel G. Jacquier A., 1986 Universal code equivalent of a yeast mitochondrial intron reading frame is expressed into *E. coli* as a specific double strand endonuclease. Cell 44: 521–533. 3004738 10.1016/0092-8674(86)90262-x

[bib17] Colleaux L. Michel-Wolwertz M. R. Matagne R. F. Dujon B., 1990 The apocytochrome *b* gene of *Chlamydomonas smithii* contains a mobile intron related to both *Saccharomyces* and *Neurospora* introns. Mol. Gen. Genet. 223: 288–296. 1701210 10.1007/BF00265065

[bib18] Dujon B., 1989 Group-I introns as mobile genetic elements - facts and mechanistic speculations—a review. Gene 82: 91–114. 2555264 10.1016/0378-1119(89)90034-6

[bib19] Edgar R. C., 2004 MUSCLE: multiple sequence alignment with high accuracy and high throughput. Nucleic Acids Res. 32: 1792–1797. 15034147 10.1093/nar/gkh340 PMC390337

[bib20] Fukami H. Chen C. A. Chiou C. Y. Knowlton N., 2007 Novel group I introns encoding a putative homing endonuclease in the mitochondrial *cox1* gene of Scleractinian corals. J. Mol. Evol. 64: 591–600. 17437148 10.1007/s00239-006-0279-4

[bib21] Goddard M. R. Burt A., 1999 Recurrent invasion and extinction of a selfish gene. Proc. Natl. Acad. Sci. USA 96: 13880–13885. 10570167 10.1073/pnas.96.24.13880 PMC24159

[bib22] Goffeau A. Barrell B. G. Bussey H. Davis R. W. Dujon B., 1996 Life with 6000 genes. Science 274: 546, 563–567. 10.1126/science.274.5287.546 8849441

[bib23] Gouy M. Guindon S. Gascuel O., 2010 SeaView version 4: a multiplatform graphical user interface for sequence alignment and phylogenetic tree building. Mol. Biol. Evol. 27: 221–224. 19854763 10.1093/molbev/msp259

[bib24] Hao W., 2010 OrgConv: detection of gene conversion using consensus sequences and its application in plant mitochondrial and chloroplast homologs. BMC Bioinformatics 11: 114. 20196863 10.1186/1471-2105-11-114 PMC2842260

[bib25] Hao W. Golding G. B., 2006 The fate of laterally transferred genes: life in the fast lane to adaptation or death. Genome Res. 16: 636–643. 16651664 10.1101/gr.4746406 PMC1457040

[bib26] Hao W. Golding G. B., 2010 Inferring bacterial genome flux while considering truncated genes. Genetics 186: 411–426. 20551435 10.1534/genetics.110.118448 PMC2940306

[bib27] Hao W. Palmer J. D., 2011 HGT turbulence: confounding phylogenetic influence of duplicative horizontal transfer and differential gene conversion. Mobile Genet. Elements 1: 256–261. 10.4161/mge.19030 PMC3337133 22545235

[bib28] Hao W. Richardson A. O. Zheng Y. Palmer J. D., 2010 Gorgeous mosaic of mitochondrial genes created by horizontal transfer and gene conversion. Proc. Natl. Acad. Sci. USA 107: 21576–21581. 21115831 10.1073/pnas.1016295107 PMC3003057

[bib29] Haugen P. Bhattacharya D., 2004 The spread of LAGLIDADG homing endonuclease genes in rDNA. Nucleic Acids Res. 32: 2049–2057. 15069127 10.1093/nar/gkh520 PMC390371

[bib30] Haugen P. Reeb V. Lutzoni F. Bhattacharya D., 2004 The evolution of homing endonuclease genes and group I introns in nuclear rDNA. Mol. Biol. Evol. 21: 129–140. 14595099 10.1093/molbev/msh005

[bib31] Haugen P. Simon D. M. Bhattacharya D., 2005 The natural history of group I introns. Trends Genet. 21: 111–119. 15661357 10.1016/j.tig.2004.12.007

[bib32] Hepburn N. J. Schmidt D. W. Mower J. P., 2012 Loss of two introns from the *Magnolia tripetala* mitochondrial *cox2* gene implicates horizontal gene transfer and gene conversion as a novel mechanism of intron loss. Mol. Biol. Evol. 29: 3111–3120. 22593225 10.1093/molbev/mss130

[bib33] Jacquier A. Dujon B., 1985 An intron-encoded protein is active in a gene conversion process that spreads an intron into a mitochondrial gene. Cell 41: 383–394. 3886163 10.1016/s0092-8674(85)80011-8

[bib34] Kellis M. Patterson N. Endrizzi M. Birren B. Lander E. S., 2003 Sequencing and comparison of yeast species to identify genes and regulatory elements. Nature 423: 241–254. 12748633 10.1038/nature01644

[bib35] Kong Y. Ma J. H. Warren K. Tsang R. S. Low D. E., 2013 Homologous recombination drives both sequence diversity and gene content variation in *Neisseria meningitidis*. Genome Biol. Evol. 5: 1611–1627. 23902748 10.1093/gbe/evt116 PMC3787668

[bib36] Kurtzman C. P., 2003 Phylogenetic circumscription of *Saccharomyces*, *Kluyveromyces* and other members of the Saccharomycetaceae, and the proposal of the new genera *Lachancea*, *Nakaseomyces*, *Naumovia*, *Vanderwaltozyma* and *Zygotorulaspora*. FEMS Yeast Res. 4: 233–245. 14654427 10.1016/S1567-1356(03)00175-2

[bib37] Lambowitz A. M. Belfort M., 1993 Introns as mobile genetic elements. Annu. Rev. Biochem. 62: 587–622. 8352597 10.1146/annurev.bi.62.070193.003103

[bib38] Lee C. K. Araki N. Sowersby D. S. Lewis L. K., 2012 Factors affecting chemical-based purification of DNA from *Saccharomyces cerevisiae*. Yeast 29: 73–80. 22134898 10.1002/yea.1918

[bib39] Loizos N. Tillier E. R. Belfort M., 1994 Evolution of mobile group I introns: recognition of intron sequences by an intron-encoded endonuclease. Proc. Natl. Acad. Sci. USA 91: 11983–11987. 7991569 10.1073/pnas.91.25.11983 PMC45360

[bib40] Loytynoja A. Goldman N., 2005 An algorithm for progressive multiple alignment of sequences with insertions. Proc. Natl. Acad. Sci. USA 102: 10557–10562. 16000407 10.1073/pnas.0409137102 PMC1180752

[bib41] Milstein D. Oliveira M. C. Martins F. M. Matioli S. R., 2008 Group I introns and associated homing endonuclease genes reveals a clinal structure for Porphyra spiralis var. amplifolia (Bangiales, Rhodophyta) along the Eastern coast of South America. BMC Evol. Biol. 8: 308. 18992156 10.1186/1471-2148-8-308 PMC2585584

[bib42] Mower J. P. Stefanovic S. Hao W. Gummow J. S. Jain K., 2010 Horizontal acquisition of multiple mitochondrial genes from a parasitic plant followed by gene conversion with host mitochondrial genes. BMC Biol. 8: 150. 21176201 10.1186/1741-7007-8-150 PMC3022774

[bib43] Mueller J. E. Smith D. Belfort M., 1996 Exon coconversion biases accompanying intron homing: battle of the nucleases. Genes Dev. 10: 2158–2166. 8804310 10.1101/gad.10.17.2158

[bib44] Nielsen H. Johansen S. D., 2009 Group I introns: Moving in new directions. RNA Biol. 6: 375–383. 19667762 10.4161/rna.6.4.9334

[bib45] Pagel M. Meade A. Barker D., 2004 Bayesian estimation of ancestral character states on phylogenies. Syst. Biol. 53: 673–684. 15545248 10.1080/10635150490522232

[bib46] Palmer S. Schildkraut E. Lazarin R. Nguyen J. Nickoloff J. A., 2003 Gene conversion tracts in *Saccharomyces cerevisiae* can be extremely short and highly directional. Nucleic Acids Res. 31: 1164–1173. 12582235 10.1093/nar/gkg219 PMC150237

[bib47] Paradis E. Claude J. Strimmer K., 2004 APE: analyses of phylogenetics and evolution in R language. Bioinformatics 20: 289–290. 14734327 10.1093/bioinformatics/btg412

[bib48] Roman J. Rubin M. N. Woodson S. A., 1999 Sequence specificity of in vivo reverse splicing of the *Tetrahymena* group I intron. RNA 5: 1–13. 9917062 10.1017/s1355838299981244 PMC1369735

[bib49] Rot C. Goldfarb I. Ilan M. Huchon D., 2006 Putative cross-kingdom horizontal gene transfer in sponge (Porifera) mitochondria. BMC Evol. Biol. 6: 71. 16972986 10.1186/1471-2148-6-71 PMC1618405

[bib50] Sanchez-Puerta M. V. Cho Y. Mower J. P. Alverson A. J. Palmer J. D., 2008 Frequent, phylogenetically local horizontal transfer of the *cox1* group I Intron in flowering plant mitochondria. Mol. Biol. Evol. 25: 1762–1777. 18524785 10.1093/molbev/msn129 PMC2727383

[bib51] Sanchez-Puerta M. V. Abbona C. C. Zhuo S. Tepe E. J. Bohs L., 2011 Multiple recent horizontal transfers of the *cox1* intron in *Solanaceae* and extended co-conversion of flanking exons. BMC Evol. Biol. 11: 277. 21943226 10.1186/1471-2148-11-277 PMC3192709

[bib52] Sellem C. H. Belcour L., 1997 Intron open reading frames as mobile elements and evolution of a group I intron. Mol. Biol. Evol. 14: 518–526. 9159929 10.1093/oxfordjournals.molbev.a025788

[bib53] Shimodaira H., 2002 An approximately unbiased test of phylogenetic tree selection. Syst. Biol. 51: 492–508. 12079646 10.1080/10635150290069913

[bib54] Shimodaira H. Hasegawa M., 2001 CONSEL: for assessing the confidence of phylogenetic tree selection. Bioinformatics 17: 1246–1247. 11751242 10.1093/bioinformatics/17.12.1246

[bib55] Skelly P. J. Maleszka R., 1991 Distribution of mitochondrial intron sequences among 21 yeast species. Curr. Genet. 19: 89–94. 2065366 10.1007/BF00326288

[bib56] Smith D. R. Keeling P. J., 2013 Gene conversion shapes linear mitochondrial genome architecture. Genome Biol. Evol. 5: 905–912. 23572386 10.1093/gbe/evt059 PMC3673629

[bib57] Spencer M. Sangaralingam A., 2009 A phylogenetic mixture model for gene family loss in parasitic bacteria. Mol. Biol. Evol. 26: 1901–1908. 19435739 10.1093/molbev/msp102

[bib58] Stamatakis A., 2006 RAxML-VI-HPC: maximum likelihood-based phylogenetic analyses with thousands of taxa and mixed models. Bioinformatics 22: 2688–2690. 16928733 10.1093/bioinformatics/btl446

[bib59] Vaughn J. C. Mason M. T. Sper-Whitis G. L. Kuhlman P. Palmer J. D., 1995 Fungal origin by horizontal transfer of a plant mitochondrial group I intron in the chimeric *CoxI* gene of *Peperomia*. J. Mol. Evol. 41: 563–572. 7490770 10.1007/BF00175814

[bib60] Wei W. McCusker J. H. Hyman R. W. Jones T. Ning Y., 2007 Genome sequencing and comparative analysis of *Saccharomyces cerevisiae* strain YJM789. Proc. Natl. Acad. Sci. USA 104: 12825–12830. 17652520 10.1073/pnas.0701291104 PMC1933262

[bib61] Wikmark O. G. Einvik C. De Jonckheere J. F. Johansen S. D., 2006 Short-term sequence evolution and vertical inheritance of the Naegleria twin-ribozyme group I intron. BMC Evol. Biol. 6: 39. 16670006 10.1186/1471-2148-6-39 PMC1464144

[bib62] Zeng Q. Bonocora R. P. Shub D. A., 2009 A free-standing homing endonuclease targets an intron insertion site in the *psbA* gene of cyanophages. Curr. Biol. 19: 218–222. 19200728 10.1016/j.cub.2008.11.069

