# Supplementary material for: Horizontal Transfer and Gene Conversion as an Important Driving Force in Shaping the Landscape of Mitochondrial Introns
Source: G3 (Bethesda). 2014 Feb 10;4(4):605–12. doi: 10.1534/g3.113.009910 (PMC4059233; doi:10.1534/g3.113.009910)
Supplement: Supporting Information [file supp_4_4_605__index.html]

Horizontal Transfer and Gene Conversion as an Important Driving Force in Shaping the Landscape of Mitochondrial Introns — Supporting Information 

# Horizontal Transfer and Gene Conversion as an Important Driving Force in Shaping the Landscape of Mitochondrial Introns

## Supporting Information for Wu and Hao, 2014

**Files in this Data Supplement:**

- Supporting Information - Figures S1-S5 and Tables S1-S2 (PDF, 1 MB)
- Figure S1 - Manually edited sequence alignment of the LSU rRNA gene containing the ω intron and HEG regions. (PDF, 1 MB)
- Figure S2 - Unfiltered sequence alignment of the LSU rRNA gene containing the ω intron and HEG regions. (PDF, 1 MB)
- Figure S3 - Log likelihood surface with different rates of gain and loss for the intron (light blue) and the HEG (red). (PDF, 214 KB)
- Figure S4 - Reconstruction of gains and losses of the intron and HEG in the evolution of the *Saccharomyces* complex. (PDF, 583 KB)
- Figure S5 - Maximum likelihood tree of the *Saccharomyces, Torulaspora* and *Lachancea* strains partial exon sequences in the LSU rRNA gene based on the sequence alignment in Figure S1. (PDF, 365 KB)
- Table S1 - GenBank accessions used in study. (PDF, 117 KB)
- Table S2 - Primer sequences used in this study. (PDF, 103 KB)
