# Supplementary material for: Horizontal Transfer and Gene Conversion as an Important Driving Force in Shaping the Landscape of Mitochondrial Introns
Source: G3 (Bethesda). 2014 Feb 10;4(4):605–12. doi: 10.1534/g3.113.009910 (PMC4059233; doi:10.1534/g3.113.009910)
Supplement: Supporting Information [file supp_g3.113.009910_009910SI.pdf]

G3· Genes | Genomes | Genetics

Horizontal Transfer and Gene Conversion as an Important Driving Force in Shaping the Landscape of Mitochondrial Introns

Baojun Wu and Weilong Hao*

Department of Biological Sciences, Wayne State University, Detroit, Michigan, USA

*Corresponding author: Wayne State University, Department of Biological Sciences, 5047 Gullen Mall, Detroit, MI 48202, Email: haow@wayne.edu

Data deposition: The sequences generated in this paper have been deposited in the GenBank (KF300887-KF300926)

DOI: 10.1534/g3.113.009910

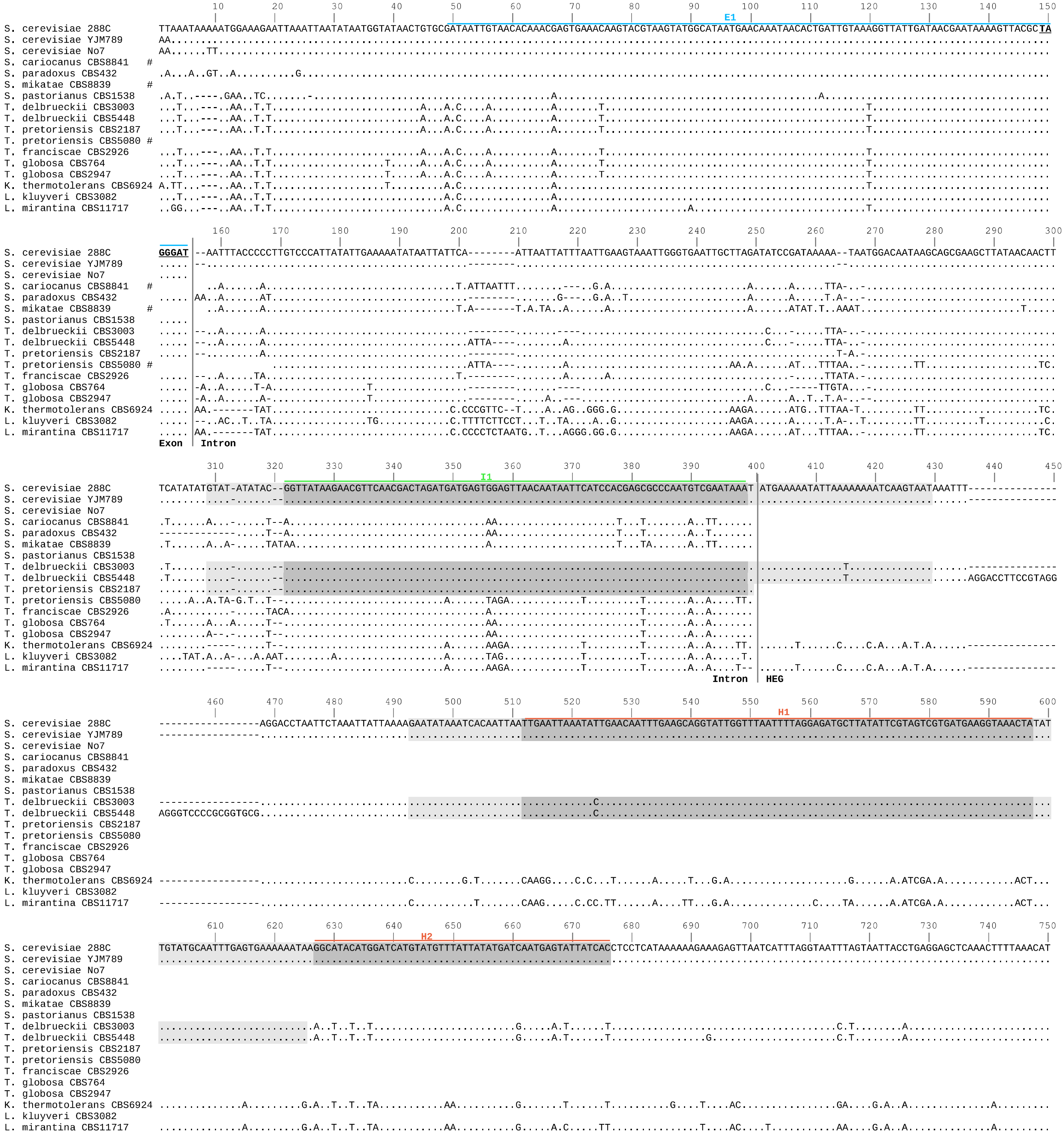
E1
I1
H1
H2
Exon
Intron
HEG
S. cerevisiae 288C
S. cerevisiae YJM789
S. cerevisiae No7
S. cariocanus CBS8841
S. paradoxus CBS432
S. mikatae CBS8839
S. pastorianus CBS1538
T. delbrueckii CBS3003
T. delbrueckii CBS5448
T. pretoriensis CBS2187
T. pretoriensis CBS5080
T. franciscae CBS2926
T. globosa CBS764
T. globosa CBS2947
K. thermotolerans CBS6924
L. kluyveri CBS3082
L. mirantina CBS11717

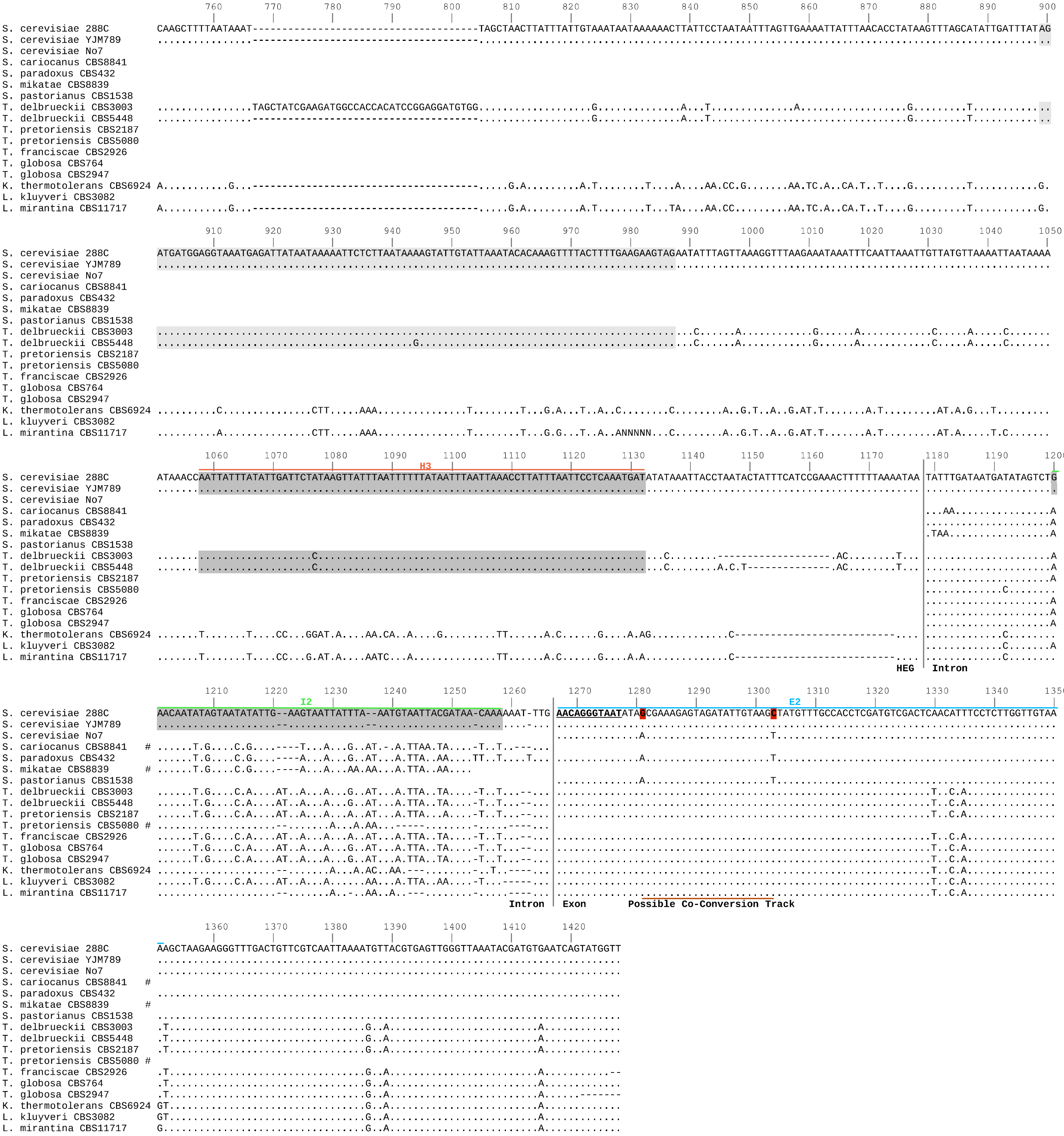

Figure S1 Manually edited sequence alignment of the LSU rRNA gene containing the ω intron and HEG regions. Dots indicate identities relative to the nucleotide sites in *S. cerevisiae 288C*, while letters show nucleotide differences. The regions shown in Figure 2 are labeled. As the regions in Figure 2 are not the exhaustive regions highly similar with *S. cerevisiae,* other regions highly similar with *S. cerevisiae* are highlighted in light gray. Boundaries for the Intron and HEG are shown. Strains with no exon sequence information are labeled with #s.

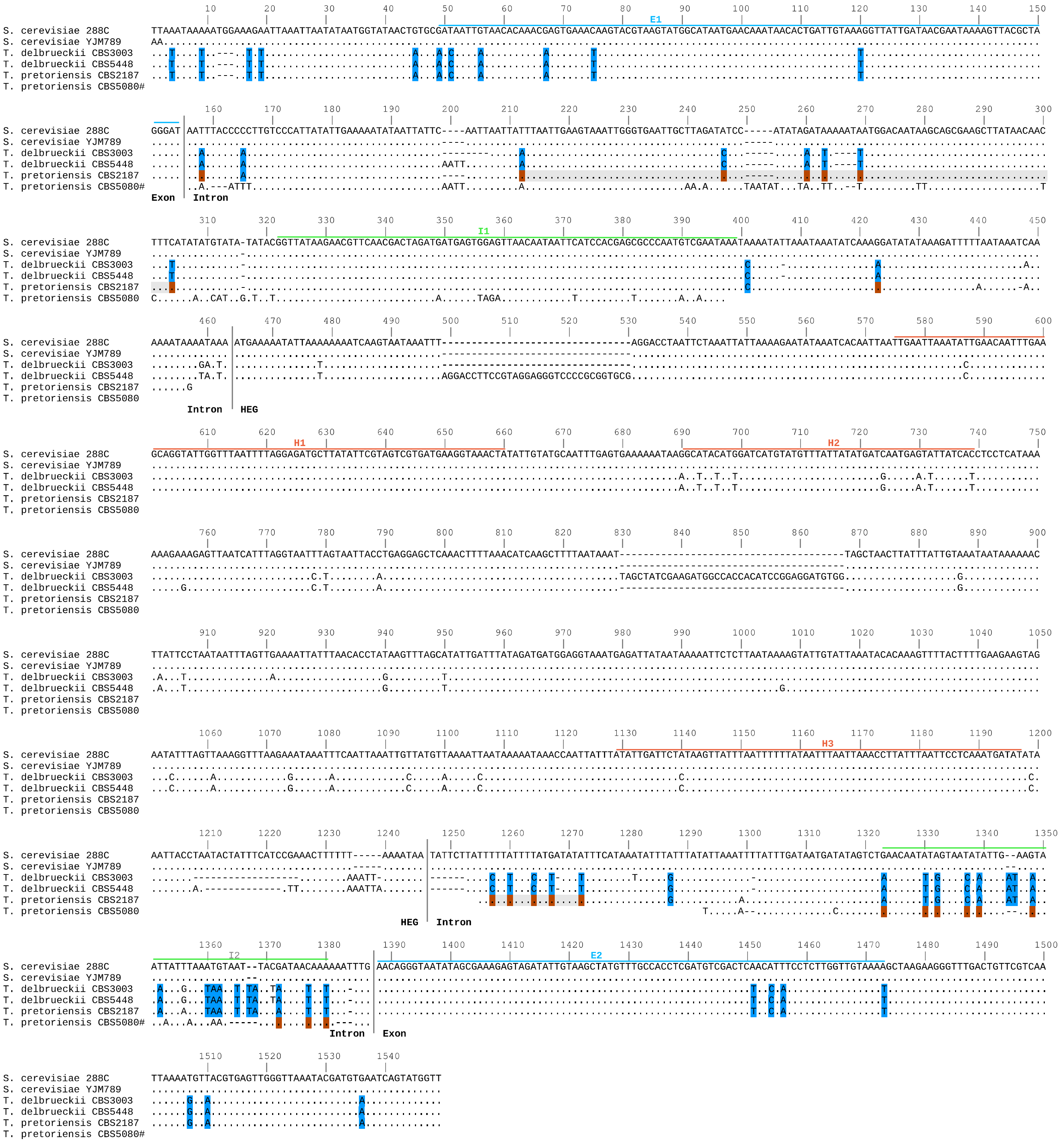

Figure S2 Unfiltered sequence alignment of the LSU rRNA gene containing the ω intron and HEG regions. No sequences were removed from the alignment. To better illustrate the mosaic structure of the *Torulaspora pretoriensis* introns, only *T. delbrueckii* and *T. pretoriensis* and two *S. cerevisiae* sequences were included. Nucleotides in *T. pretoriensis* identical to *T. delbrueckii* but different from *S. cerevisiae* are highlighted in blue, while the ones identical to *S. cerevisiae* but different from *T. delbrueckii* are in red. Regions in *T. pretoriensis* CBS2187 with consecutive nucleotides identical to *S. cerevisiae* but different from *T. delbrueckii* are highlighted in gray. *T. pretoriensis* CBS5080 does not have exon sequence information and is labeled with #.

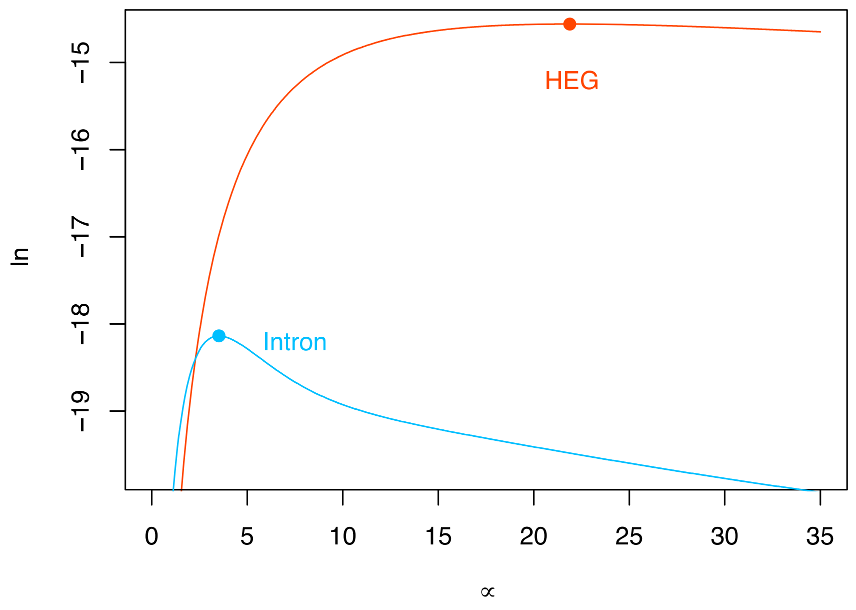

Figure S3 Log likelihood surface with different rates of gain and loss for the intron (light blue) and the HEG (red). The likelihood values were calculated based on the data shown in Figure 2. The filled circles are the estimated turnover rates giving the maximum (peak) likelihood value on each dataset.

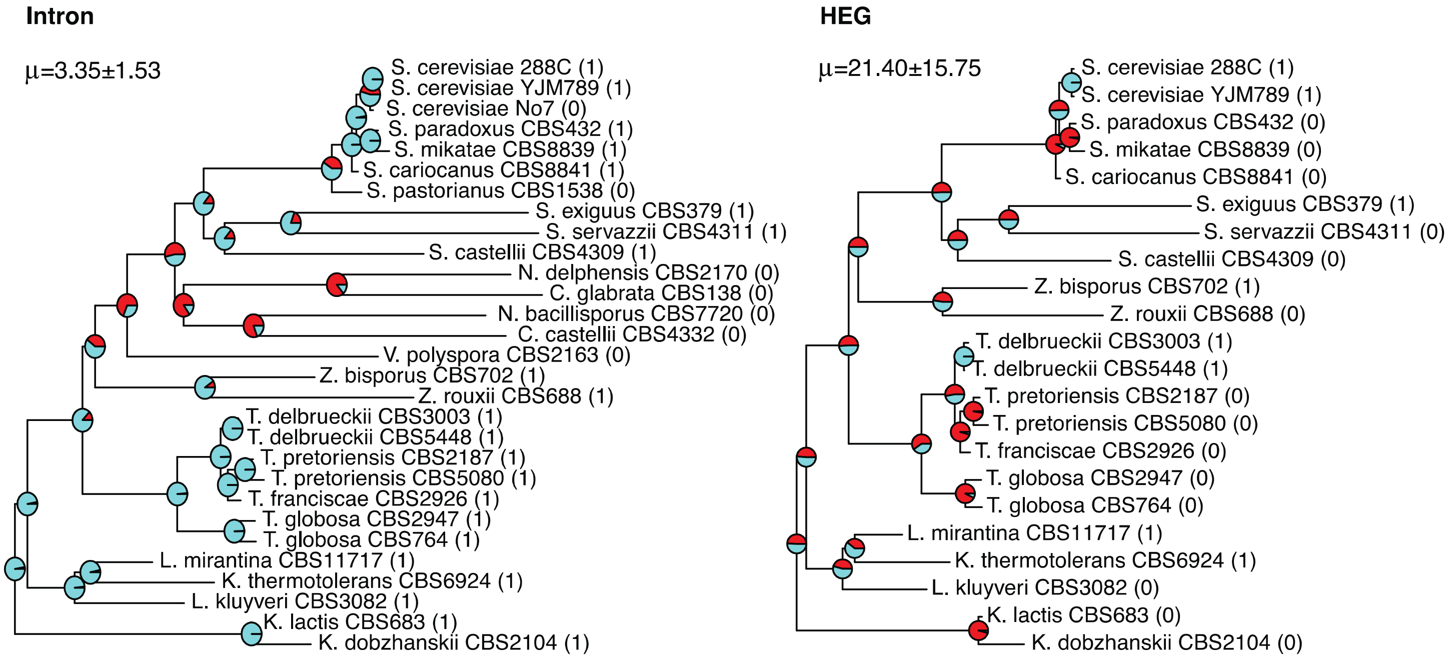

Figure S4 Reconstruction of gains and losses of the intron and HEG in the evolution of the *Saccharomyces* complex. The branch lengths were optimized using the obtained four regions, ITS1-5.8S-ITS2, 26S rRNA D1/D2, mitochondrial small subunit rRNA and *cox2* based on the topology published in Kurtzman (2003). Pie charts illustrate the relative likelihoods (local estimators) of the two possible states (presence or absence) of the intron or HEG at each ancestral node. The rate (μ) of gain and loss was estimated separately for the intron and HEG.

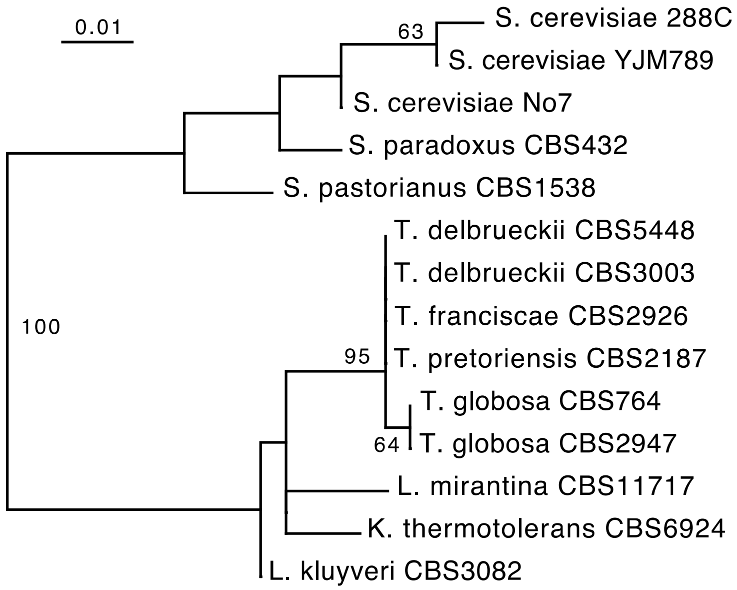

Figure S5 Maximum likelihood tree of the *Saccharomyces*, *Torulaspora* and *Lachancea* strains partial exon sequences in the LSU rRNA gene based on the sequence alignment in Supplementary Figure S1. Bootstrap values when >60% are shown. Although the *Lachancea* clade is shown as paraphyletic, the deep relationship is clearly resolved as the three clades are well separated. This phylogeny was not significantly different from the phylogeny constructed for the same strains based on the concatenated sequences of ITS1-5.8S-ITS2, 26S rRNA D1/D2, mitochondrial SSU rRNA and *cox2* in the AU test.

Table S1 GenBank accessions used in study.

Species/Strain Names	ITS	26SD1/D2	COX2	SSU	LSU
Candida castellii CBS4332	AY046196	U69876	AF442255	AF442329	FM995165
Candida glabrata CBS138	AY046165	U44808	AF442225	AF442299	AJ511533
Kluyveromyces dobzhanskii CBS2104	AY046215	U69575	AF442272	AF442348	AJ229054
Kluyveromyces lactis CBS683	AY046213	U94922	AF442270	AF442346	AJ229055
Kluyveromyces thermotolerans CBS6924	AJ229073	-	HE983610	HE983610	HE983610
Lachancea kluyveri CBS3082	AY046209	U68552	AF442266	AF442342	HE664110
Lachancea mirantina CBS11717	-	FJ666084	HE983613	HE983613	HE983613
Nakaseomyces bacillisporus CBS7720	AY046195	U69583	AF442254	AF442328	FM995166
Nakaseomyces delphensis CBS2170	AY046166	U69576	AF442226	AF442300	FM995164
Saccharomyces castellii CBS4309	HE576754	U68557	AF442239	AF442313	AJ229049
Saccharomyces cariocanus CBS8841	AY046147	AF398478	AF442207	AF442282	AJ229045
Saccharomyces cerevisiae 288C	BK006945	BK006945	AJ011856	AJ011856	AJ011856
Saccharomyces cerevisiae No7	BABQ01000122	BABQ01000122	AP012028	AP012028	AP012028
Saccharomyces cerevisiae YJM789	JQ277730	JQ277730	EU004203	EU004203	EU004203
Saccharomyces exiguus CBS379	AY046170	U68553	AF442230	AF442304	AJ229047
Saccharomyces mikatae CBS8839	AY046149	AF398479	AF442209	AF442284	AJ229048
Saccharomyces paradoxus CBS432	AY046148	U68555	AF442208	AF442283	JQ862335
Saccharomyces pastorianus CBS1538	AY046151	AY048172	AF442212	AF442287	EU852811[b]
Saccharomyces servazzii CBS4311	AY046153	U68558	AF442213	AF442288	AJ430679
Torulaspora delbrueckii CBS133	KF300899[a]	KF300893[a]	KF300923[a]	-	KF300904[a]
Torulaspora delbrueckii CBS404	-	-	-	-	AJ229052
Torulaspora delbrueckii CBS2734	KF300897[a]	KF300891[a]	KF300920[a]	KF300914[a]	KF300907[a]
Torulaspora delbrueckii CBS3003	-	KF300894[a]	KF300925[a]	-	KF300903[a]
Torulaspora delbrueckii CBS5448	KF300900[a]	-	KF300924[a]	KF300917[a]	KF300902[a]
Torulaspora delbrueckii CBS6786	-	-	KF300921[a]	KF300915[a]	KF300906[a]
Torulaspora delbrueckii CBS6991	KF300898[a]	KF300892[a]	KF300922[a]	KF300916[a]	KF300905[a]
Torulaspora globosa CBS764	KF300896[a]	KF300889[a]	KF300919[a]	KF300913[a]	KF300909[a]
Torulaspora globosa CBS2947	KF300895[a]	KF300888[a]	KF300926[a]	-	KF300910[a]
Torulaspora franciscae CBS2926	AY046186	KF300890[a]	AF442245	AF442319	KF300908[a]
Torulaspora pretoriensis CBS5080	AJ229066	-	-	-	AJ229051
Torulaspora pretoriensis CBS2187	KF300901[a]	KF300887[a]	KF300918[a]	KF300912[a]	KF300911[a]
Vanderwaltozyma polyspora CBS2163	AY046182	U68548	AF442241	AF442315	AM698041
Zygosaccharomyces bisporus CBS702	AY046192	U72162	AF442251	AF442325	AJ229056
Zygosaccharomyces rouxii CBS688	AJ229071	AB302812[c]	AB302806[d]	AF442322[d]	AJ229050

[a]Sequences (KF300887-KF300926) were generated in this study
[b] from *Saccharomyces pastorianus* Weihenstephan 34/70
[c] from *Zygosaccharomyces rouxii* CBS8000
[d] from *Zygosaccharomyces rouxii* CBS732.

Table S2 Primer sequences used in this study.

Region (Species)	Forward primer	Reverse primer	Designed
Intron+exon2	5'GATAACGAATAAAAGTTACGCTAGGG3'	5'CTTCAGCAGATAGGAACCATACTG3'	Ref. [S1]
Exon1 +intron 1 (*Torulaspora delbrueckii)*	5'CAATCTCTAATTGGTAGTTTTGATGG3'	5'GTTTACCTTCATCACGACTACGAAT3'	This study
Exon1 +intron 1 (other *Torulaspora*)	5'CAATCTCTAATTGGTAGTTTTGATGG3'	5'ATATCTACTCTTTCGCTATATTACCCT3'	This study
Intron2 +Exon2 (*Torulaspora delbrueckii)*	5'GTTTACCTTCATCACGACTACGAAT 3'		This study
mtSSU	5'CGAAAGATTGATCCAGTTA3'	5'GCGGATTATCGAATTAAATAAC3'	Ref. [S2]
COXII	5'AGTATCATGATTATTATTTACAATTGT3'	5'CCATAGAATACACCTTCTCTTTG3'	This study
26SRNA	5'GCATATCAATAAGCGGAGGAAAAG3'	5'GGTCCGTGTTTCAAGACGG3'	Ref. [S2]
ITS	5'TCCGTAGGTGAACCTGCGG3'	5'TCCTCCGCTTATTGATATGC3'	Ref. [S3]

S1. Goddard, M.R., and Burt, A. (1999). Recurrent invasion and extinction of a selfish gene. Proc Natl Acad Sci U S A. *96*, 13880-13885.

S2. Kurtzman, C.P., and Robnett, C.J. (2003). Phylogenetic relationships among yeasts of the '*Saccharomyces* complex' determined from multigene sequence analyses. FEMS Yeast Res. *3*, 417-432.

S3. White, T.J., Bruns, T., Lee, S., and Taylor, J. (1990). Amplification and direct sequencing of fungal ribosomal RNA genes for phylogenetics. In PCR protocols: a guide to methods and applications, Innis, M.A., Gelfand, D.H., Sninsky, J.J., and White, T.J., eds. (New York: Academic Press), pp.315-322.
