# Supplementary material for: Horizontal Transfer and Gene Conversion as an Important Driving Force in Shaping the Landscape of Mitochondrial Introns
Source: G3 (Bethesda). 2014 Feb 10;4(4):605–12. doi: 10.1534/g3.113.009910 (PMC4059233; doi:10.1534/g3.113.009910)
Supplement: Supporting Information [file supp_g3.113.009910_FigureS1.pdf]

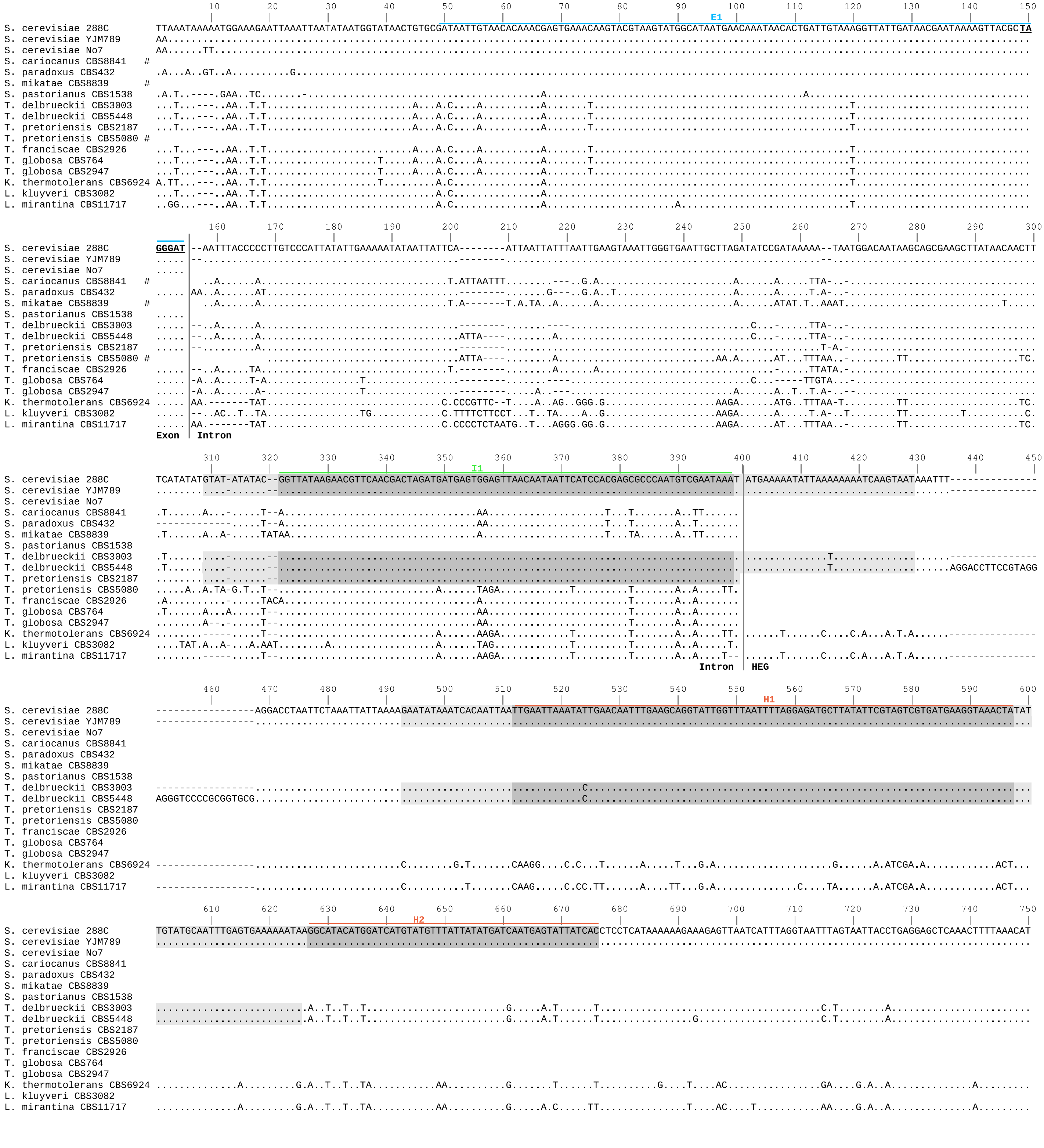
E1
I1
H1
H2
Exon
Intron
HEG
S. cerevisiae 288C
S. cerevisiae YJM789
S. cerevisiae No7
S. cariocanus CBS8841
S. paradoxus CBS432
S. mikatae CBS8839
S. pastorianus CBS1538
T. delbrueckii CBS3003
T. delbrueckii CBS5448
T. pretoriensis CBS2187
T. pretoriensis CBS5080
T. franciscae CBS2926
T. globosa CBS764
T. globosa CBS2947
K. thermotolerans CBS6924
L. kluyveri CBS3082
L. mirantina CBS11717

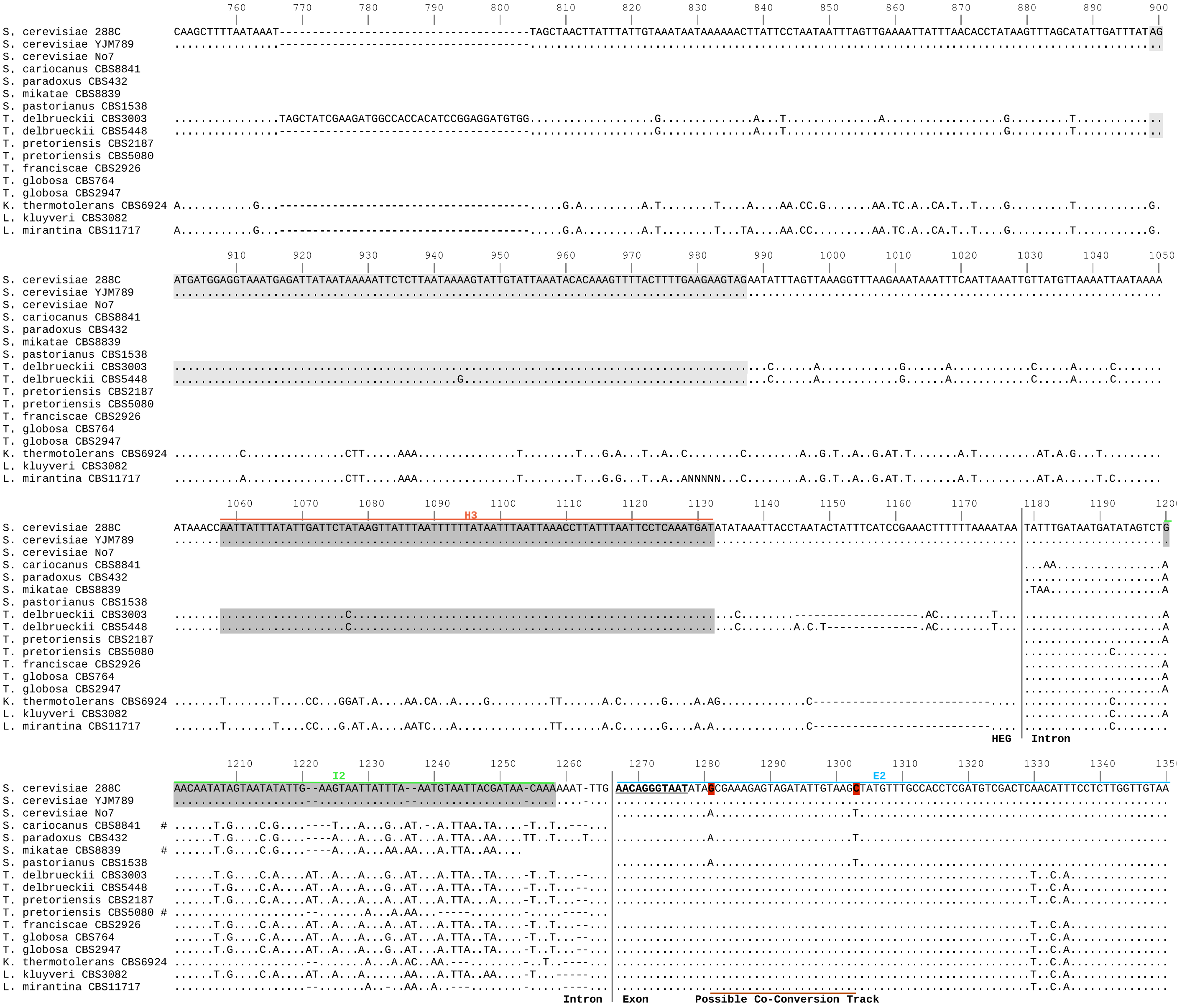

Figure S1 Manually edited sequence alignment of the LSU rRNA gene containing the ω intron and HEG regions. Dots indicate identities relative to the nucleotide sites in *S. cerevisiae 288C*, while letters show nucleotide differences. The regions shown in Figure 2 are labeled. As the regions in Figure 2 are not the exhaustive regions highly similar with *S. cerevisiae,* other regions highly similar with *S. cerevisiae* are highlighted in light gray. Boundaries for the Intron and HEG are shown. Strains with no exon sequence information are labeled with #s.
