# Supplementary material for: Horizontal Transfer and Gene Conversion as an Important Driving Force in Shaping the Landscape of Mitochondrial Introns
Source: G3 (Bethesda). 2014 Feb 10;4(4):605–12. doi: 10.1534/g3.113.009910 (PMC4059233; doi:10.1534/g3.113.009910)
Supplement: Supporting Information [file supp_g3.113.009910_FigureS2.pdf]

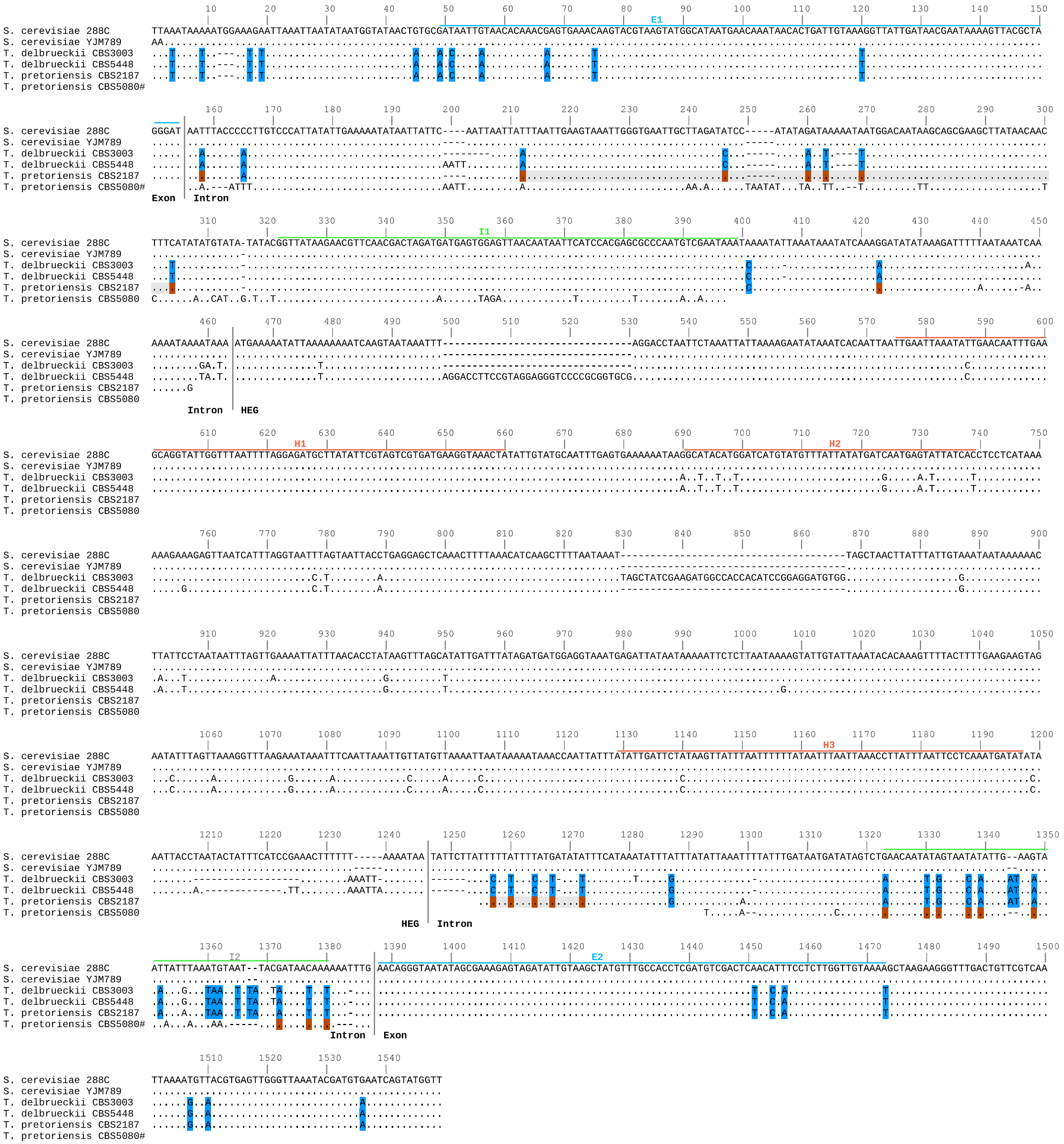

Figure S2 Unfiltered sequence alignment of the LSU rRNA gene containing the ω intron and HEG regions. No sequences were removed from the alignment. To better illustrate the mosaic structure of the *Torulaspora pretoriensis* introns, only *T. delbrueckii* and *T. pretoriensis* and two *S. cerevisiae* sequences were included. Nucleotides in *T. pretoriensis* identical to *T. delbrueckii* but different from *S. cerevisiae* are highlighted in blue, while the ones identical to *S. cerevisiae* but different from *T. delbrueckii* are in red. Regions in *T. pretoriensis* CBS2187 with consecutive nucleotides identical to *S. cerevisiae* but different from *T. delbrueckii* are highlighted in gray. *T. pretoriensis* CBS5080 does not have exon sequence information and is labeled with #.
