# Supplementary material for: Horizontal Transfer and Gene Conversion as an Important Driving Force in Shaping the Landscape of Mitochondrial Introns
Source: G3 (Bethesda). 2014 Feb 10;4(4):605–12. doi: 10.1534/g3.113.009910 (PMC4059233; doi:10.1534/g3.113.009910)
Supplement: Supporting Information [file supp_g3.113.009910_FigureS3.pdf]

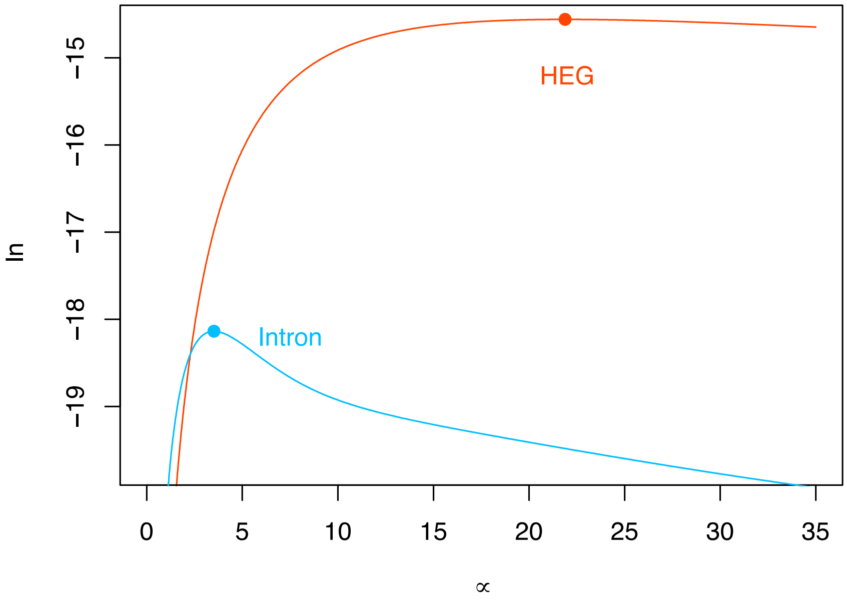

Figure S3 Log likelihood surface with different rates of gain and loss for the intron (light blue) and the HEG (red). The likelihood values were calculated based on the data shown in Figure 2. The filled circles are the estimated turnover rates giving the maximum (peak) likelihood value on each dataset.
