# Supplementary material for: Horizontal Transfer and Gene Conversion as an Important Driving Force in Shaping the Landscape of Mitochondrial Introns
Source: G3 (Bethesda). 2014 Feb 10;4(4):605–12. doi: 10.1534/g3.113.009910 (PMC4059233; doi:10.1534/g3.113.009910)
Supplement: Supporting Information [file supp_g3.113.009910_FigureS4.pdf]

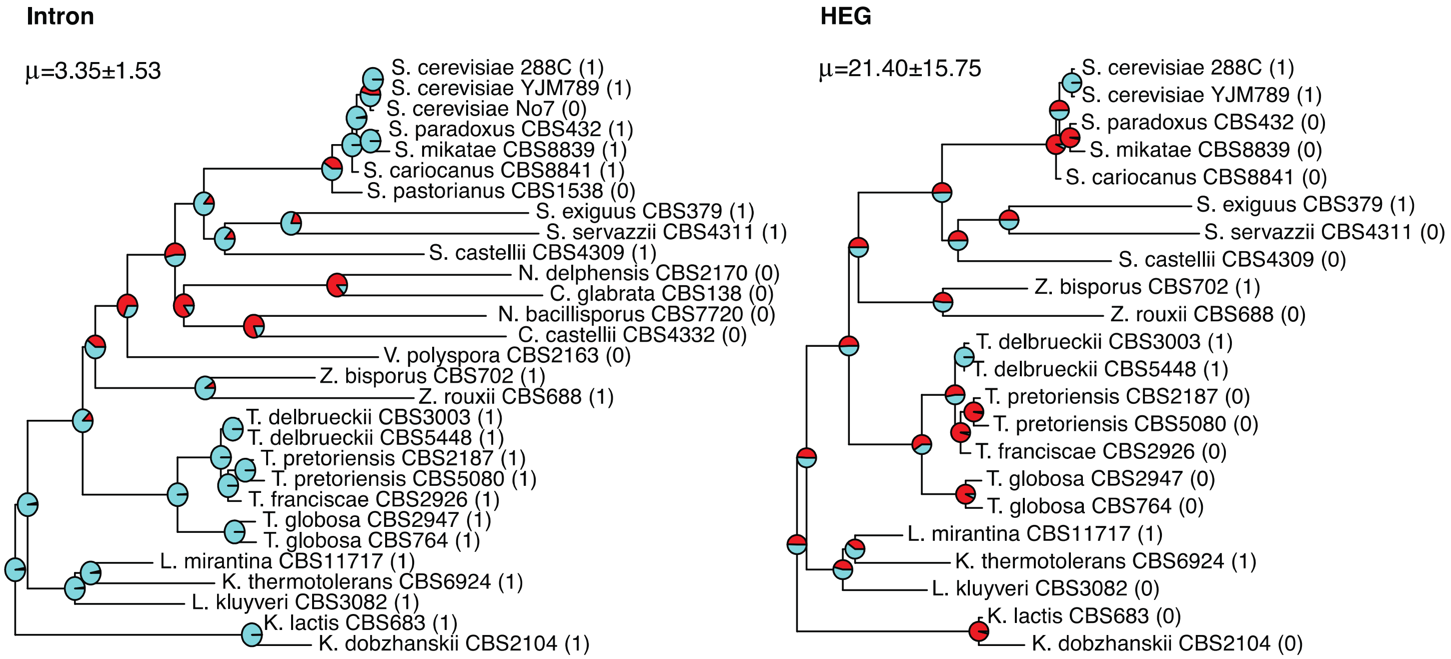

Figure S4 Reconstruction of gains and losses of the intron and HEG in the evolution of the *Saccharomyces* complex. The branch lengths were optimized using the obtained four regions, ITS1-5.8S-ITS2, 26S rRNA D1/D2, mitochondrial small subunit rRNA and *cox2* based on the topology published in Kurtzman (2003). Pie charts illustrate the relative likelihoods (local estimators) of the two possible states (presence or absence) of the intron or HEG at each ancestral node. The rate (μ) of gain and loss was estimated separately for the intron and HEG.
