# Supplementary material for: Horizontal Transfer and Gene Conversion as an Important Driving Force in Shaping the Landscape of Mitochondrial Introns
Source: G3 (Bethesda). 2014 Feb 10;4(4):605–12. doi: 10.1534/g3.113.009910 (PMC4059233; doi:10.1534/g3.113.009910)
Supplement: Supporting Information [file supp_g3.113.009910_FigureS5.pdf]

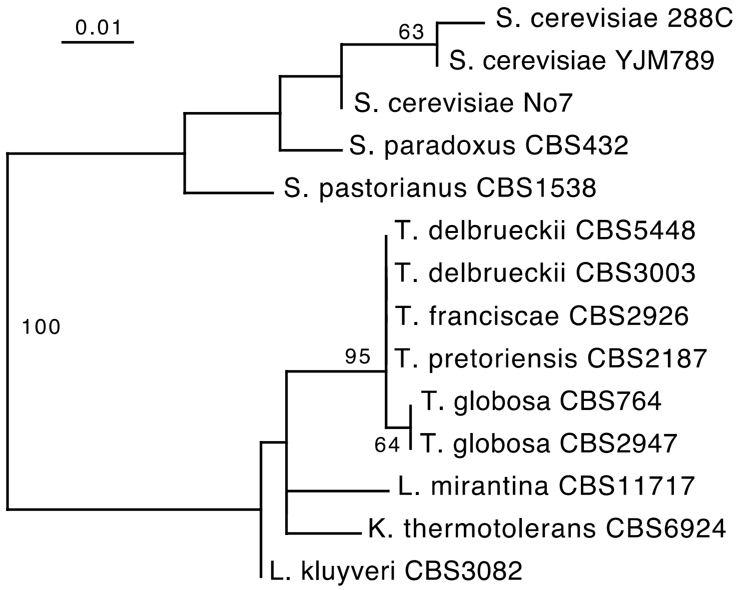

Figure S5 Maximum likelihood tree of the *Saccharomyces*, *Torulaspora* and *Lachancea* strains partial exon sequences in the LSU rRNA gene based on the sequence alignment in Supplementary Figure S1. Bootstrap values when >60% are shown. Although the *Lachancea* clade is shown as paraphyletic, the deep relationship is clearly resolved as the three clades are well separated. This phylogeny was not significantly different from the phylogeny constructed for the same strains based on the concatenated sequences of ITS1-5.8S-ITS2, 26S rRNA D1/D2, mitochondrial SSU rRNA and *cox2* in the AU test.
