# Supplementary material for: Horizontal Transfer and Gene Conversion as an Important Driving Force in Shaping the Landscape of Mitochondrial Introns
Source: G3 (Bethesda). 2014 Feb 10;4(4):605–12. doi: 10.1534/g3.113.009910 (PMC4059233; doi:10.1534/g3.113.009910)
Supplement: Supporting Information [file supp_g3.113.009910_TableS1.pdf]

Table S1 GenBank accessions used in study.

Species/Strain Names	ITS	26SD1/D2	COX2	SSU	LSU
Candida castellii CBS4332	AY046196	U69876	AF442255	AF442329	FM995165
Candida glabrata CBS138	AY046165	U44808	AF442225	AF442299	AJ511533
Kluyveromyces dobzhanskii CBS2104	AY046215	U69575	AF442272	AF442348	AJ229054
Kluyveromyces lactis CBS683	AY046213	U94922	AF442270	AF442346	AJ229055
Kluyveromyces thermotolerans CBS6924	AJ229073	-	HE983610	HE983610	HE983610
Lachancea kluyveri CBS3082	AY046209	U68552	AF442266	AF442342	HE664110
Lachancea mirantina CBS11717	-	FJ666084	HE983613	HE983613	HE983613
Nakaseomyces bacillisporus CBS7720	AY046195	U69583	AF442254	AF442328	FM995166
Nakaseomyces delphensis CBS2170	AY046166	U69576	AF442226	AF442300	FM995164
Saccharomyces castellii CBS4309	HE576754	U68557	AF442239	AF442313	AJ229049
Saccharomyces cariocanus CBS8841	AY046147	AF398478	AF442207	AF442282	AJ229045
Saccharomyces cerevisiae 288C	BK006945	BK006945	AJ011856	AJ011856	AJ011856
Saccharomyces cerevisiae No7	BABQ01000122	BABQ01000122	AP012028	AP012028	AP012028
Saccharomyces cerevisiae YJM789	JQ277730	JQ277730	EU004203	EU004203	EU004203
Saccharomyces exiguus CBS379	AY046170	U68553	AF442230	AF442304	AJ229047
Saccharomyces mikatae CBS8839	AY046149	AF398479	AF442209	AF442284	AJ229048
Saccharomyces paradoxus CBS432	AY046148	U68555	AF442208	AF442283	JQ862335
Saccharomyces pastorianus CBS1538	AY046151	AY048172	AF442212	AF442287	EU852811[b]
Saccharomyces servazzii CBS4311	AY046153	U68558	AF442213	AF442288	AJ430679
Torulaspora delbrueckii CBS133	KF300899[a]	KF300893[a]	KF300923[a]	-	KF300904[a]
Torulaspora delbrueckii CBS404	-	-	-	-	AJ229052
Torulaspora delbrueckii CBS2734	KF300897[a]	KF300891[a]	KF300920[a]	KF300914[a]	KF300907[a]
Torulaspora delbrueckii CBS3003	-	KF300894[a]	KF300925[a]	-	KF300903[a]
Torulaspora delbrueckii CBS5448	KF300900[a]	-	KF300924[a]	KF300917[a]	KF300902[a]
Torulaspora delbrueckii CBS6786	-	-	KF300921[a]	KF300915[a]	KF300906[a]
Torulaspora delbrueckii CBS6991	KF300898[a]	KF300892[a]	KF300922[a]	KF300916[a]	KF300905[a]
Torulaspora globosa CBS764	KF300896[a]	KF300889[a]	KF300919[a]	KF300913[a]	KF300909[a]
Torulaspora globosa CBS2947	KF300895[a]	KF300888[a]	KF300926[a]	-	KF300910[a]
Torulaspora franciscae CBS2926	AY046186	KF300890[a]	AF442245	AF442319	KF300908[a]
Torulaspora pretoriensis CBS5080	AJ229066	-	-	-	AJ229051
Torulaspora pretoriensis CBS2187	KF300901[a]	KF300887[a]	KF300918[a]	KF300912[a]	KF300911[a]
Vanderwaltozyma polyspora CBS2163	AY046182	U68548	AF442241	AF442315	AM698041
Zygosaccharomyces bisporus CBS702	AY046192	U72162	AF442251	AF442325	AJ229056
Zygosaccharomyces rouxii CBS688	AJ229071	AB302812[c]	AB302806[d]	AF442322[d]	AJ229050

[a]Sequences (KF300887-KF300926) were generated in this study
[b] from *Saccharomyces pastorianus* Weihenstephan 34/70
[c] from *Zygosaccharomyces rouxii* CBS8000
[d] from *Zygosaccharomyces rouxii* CBS732.
