# Supplementary material for: Horizontal Transfer and Gene Conversion as an Important Driving Force in Shaping the Landscape of Mitochondrial Introns
Source: G3 (Bethesda). 2014 Feb 10;4(4):605–12. doi: 10.1534/g3.113.009910 (PMC4059233; doi:10.1534/g3.113.009910)
Supplement: Supporting Information [file supp_g3.113.009910_TableS2.pdf]

Table S2 Primer sequences used in this study.

Region (Species)	Forward primer	Reverse primer	Designed
Intron+exon2	5'GATAACGAATAAAAGTTACGCTAGGG3'	5'CTTCAGCAGATAGGAACCATACTG3'	Ref. [S1]
Exon1 +intron 1 (*Torulaspora delbrueckii)*	5'CAATCTCTAATTGGTAGTTTTGATGG3'	5'GTTTACCTTCATCACGACTACGAAT3'	This study
Exon1 +intron 1 (other *Torulaspora*)	5'CAATCTCTAATTGGTAGTTTTGATGG3'	5'ATATCTACTCTTTCGCTATATTACCCT3'	This study
Intron2 +Exon2 (*Torulaspora delbrueckii)*	5'GTTTACCTTCATCACGACTACGAAT 3'		This study
mtSSU	5'CGAAAGATTGATCCAGTTA3'	5'GCGGATTATCGAATTAAATAAC3'	Ref. [S2]
COXII	5'AGTATCATGATTATTATTTACAATTGT3'	5'CCATAGAATACACCTTCTCTTTG3'	This study
26SRNA	5'GCATATCAATAAGCGGAGGAAAAG3'	5'GGTCCGTGTTTCAAGACGG3'	Ref. [S2]
ITS	5'TCCGTAGGTGAACCTGCGG3'	5'TCCTCCGCTTATTGATATGC3'	Ref. [S3]

S1. Goddard, M.R., and Burt, A. (1999). Recurrent invasion and extinction of a selfish gene. Proc Natl Acad Sci U S A. *96*, 13880-13885.

S2. Kurtzman, C.P., and Robnett, C.J. (2003). Phylogenetic relationships among yeasts of the '*Saccharomyces* complex' determined from multigene sequence analyses. FEMS Yeast Res. *3*, 417-432.

S3. White, T.J., Bruns, T., Lee, S., and Taylor, J. (1990). Amplification and direct sequencing of fungal ribosomal RNA genes for phylogenetics. In PCR protocols: a guide to methods and applications, Innis, M.A., Gelfand, D.H., Sninsky, J.J., and White, T.J., eds. (New York: Academic Press), pp.315-322.
